# Geographical Factor Influences the Metabolite Distribution of House Edible Bird's Nests in Malaysia

**DOI:** 10.3389/fnut.2021.658634

**Published:** 2021-06-28

**Authors:** Shi-Ruo Tong, Ting-Hun Lee, Soon-Keng Cheong, Yang-Mooi Lim

**Affiliations:** ^1^Centre for Cancer Research, Faculty of Medicine and Health Sciences, Universiti Tunku Abdul Rahman, Cheras, Malaysia; ^2^Bioprocess and Polymer Engineering, Faculty of Engineering, School of Chemical & Energy Engineering, Universiti Teknologi Malaysia, Johor Bahru, Malaysia; ^3^Department of Medicine, Faculty of Medicine and Health Sciences, Universiti Tunku Abdul Rahman, Cheras, Malaysia; ^4^Department of Pre-clinical Sciences, Faculty of Medicine and Health Sciences, Universiti Tunku Abdul Rahman, Cheras, Malaysia

**Keywords:** house edible bird's nest, metabolite profiling, hierarchical clustering analysis, geographical distribution, liquid chromatography-mass spectrometry

## Abstract

**Background:** Edible Bird's Nest (EBN) is famously consumed as a food tonic for its high nutritional values with numerous recuperative and therapeutic properties. EBN is majority exploited from swiftlet houses but the differences in terms of metabolite distribution between the production site of house EBN is not yet fully understood. Therefore, this study was designed to identify the metabolite distribution and to determine the relationship pattern for the metabolite distribution of house EBNs from different locations in Malaysia.

**Methods:** The differences of metabolite distribution in house EBN were studied by collecting the samples from 13 states in Malaysia. An extraction method of eHMG was acquired to extract the metabolites of EBN and was subjected to non-targeted metabolite profiling *via* liquid chromatography-mass spectrometry (LC-MS). Unsupervised multivariate analysis and Venn diagram were used to explore the relationship pattern among the house EBNs in Malaysia. The geographical distribution surrounded the swiftlet house was investigated to understand its influences on the metabolite distribution.

**Results:** The hierarchical clustering analysis (HCA) combined with correlation coefficient revealed the differences between the house EBNs in Malaysia with four main clusters formation. The metabolites distribution among these clusters was unique with their varied combination of geographical distribution. Cluster 1 grouped EBNs from Selangor, Melaka, Negeri Sembilan, Terengganu which geographically distributed with major oil palm field in township; Cluster 2 included Perak and Sarawak with high distribution of oil palm in higher altitude; Cluster 3 included Perlis, Kelantan, Kedah, Penang from lowland of paddy field in village mostly and Cluster 4 grouped Sabah, Pahang, Johor which are majorly distributed with undeveloped hills. The metabolites which drove each cluster formation have happened in a group instead of individual key metabolite. The major metabolites that characterised Cluster 1 were fatty acids, while the rest of the clusters were peptides and secondary metabolites.

**Conclusion:** The metabolite profiling conducted in this study was able to discriminate the Malaysian house EBNs based on metabolites distribution. The factor that most inferences the differences of house EBNs were the geographical distribution, in which geographical distribution affects the distribution of insect and the diet of swiftlet.

## Introduction

Birds build their own nest with different kinds of material to lay eggs and protect the nestlings. Interestingly, the swiftlet from *Aerodramus* and *Collocalia* families build their nest with its own glutinous translucent filament strand of saliva (1). The nest made from the saliva of swiftlet is thought to be a food tonic delicacy and it has been eaten for its recuperative effects since the Tang dynasty (618–907 A.D.) in China (2–4). Therefore, these nests produced from *Aerodramus* and *Collocalia* swiftlets are regarded as “Edible Bird's Nest” (EBN).

EBN has been demonstrated for its therapeutic properties scientifically on suppressing the virus, oxidative stress and inflammation effect. Besides, EBN was also able to strengthen bones, reduce the thinning of the dermal layer, possess neuroprotective properties and proliferative effects on human adipose-derived stem cells and corneal keratocytes (5–14). EBN contains high nutritional value, in which it composes mostly of protein (24.4 – 66.9%), followed by carbohydrates (8.5 – 58.2%) and fats with the lowest percentage (0.01 – 2.0%) (1, 2, 15). Therefore, the consumption of nutritious EBN is famous till today for its various recuperative and proven therapeutic effects. Though the nutritional and therapeutic values of EBN have been much reported, the metabolites found in EBN that contribute to the abovementioned therapeutic properties have not been fully studied.

The value of the EBN has prompted with greater demand over time which leads to the occurrence of over-exploitation from their natural cave breeding sites, despite laws and regulations have been implemented (16). Thus, the depletion of the swiftlet population in the natural cave had happened (17). To conserve the swiftlet population while fulfilling the demands for the community, purpose build houses that mimicking the macro- and micro-environment of the swiftlet natural breeding sites have emerged. The purposed build house is often termed as swiftlet houses. Though swiftlet inhabited in the swiftlet houses, they remained their natural self-feeding behaviour at the environment around the swiftlet house. The supply of EBN today is obtained mainly from the swiftlet house farming and termed as “house EBN.”

Due to the geographical distribution of swiftlet, Indonesia is the country with the highest production of EBN accounting 85% of the world market, followed by Malaysia and Thailand (1, 18). Therefore, the differences among the EBNs from different production sites (natural cave and swiftlet house) and geographical origin (countries) have aroused interest and been massively studied in the field (4, 19–22). However, the differences of EBN obtained from different swiftlet houses at various locations in Malaysia have not been comprehensively studied, especially with the inclusion of secondary metabolites. Therefore, in this study, non-targeted metabolite profiling analysis was used to determine the differences of metabolite distribution between the house EBNs from all the 13 states in Malaysia. On the other hand, unsupervised multivariate analysis was adopted to investigate the pattern of the relationship among the house EBNs in Malaysia (20, 23).

## Materials and Methods

### Materials and Reagents

LC-MS grade formic acid and acetonitrile were purchased from Fisher Scientific (Waltham, MA, USA). Deionized water was obtained from a Barnstead GenPure water purification system (Thermo Fisher Scientific Inc, Waltham, MA, USA).

**Figure 1:**
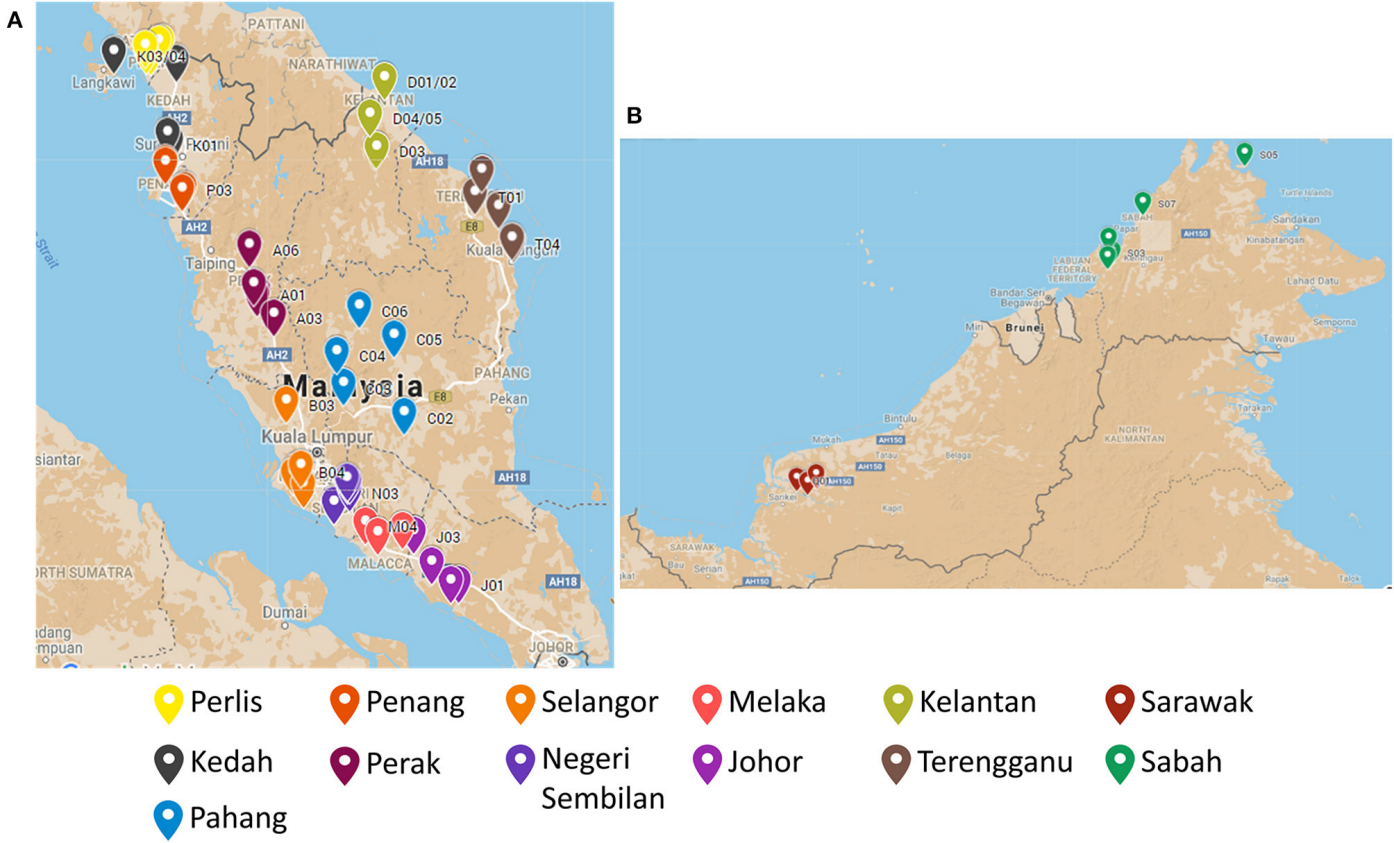
The location distribution of the EBN sampling point across **(A)** Peninsular Malaysia and **(B)** East Malaysia.

There were in total of 65 EBN samples collected from all the 13 states in Malaysia. To describe in detail, there were five biological samples collected from different swiftlet houses in each state. All the samples were originated from *Aerodramus fuciphagus* swiftlet. The location distribution of the EBN sample is illustrated in Figure 1 and detailed in Supplementary Table 1.

### Sample Preparation

All the raw EBN samples were soaked in the distilled water for an hour to loosen the laminar of saliva for subsequent feathers and impurities picking and removing. Cleaned EBN was dried in the oven at 50–55°C overnight. Dried and cleaned EBN was pulverised with mortar and pestles, followed by screening through 0.4 mm mesh size. The ground EBNs were sealed in an airtight bottle and kept at room temperature.

The metabolites of the pulverised EBN were extracted with non-disclosure eHMG method prepared by the School of Chemical and Energy Engineering at Universiti Teknologi Malaysia (UTM) (7, 24). The general extraction method was the EBN suspended in distilled deionized water at a ratio of 1:5 (w/v) and eluted for 24 h at 4°C. The mixture was boiled for an hour followed by centrifugation at 2,268 *g* (3.75 rpm) for 15 min. The supernatant was collected and immediately brought to boil. The sample solution was then flushed with nitrogen gas for a few seconds and kept at 4°C prior analysis.

### Quadrupole Time-of-Flight Liquid Chromatography-Mass Spectrometry (QTOF LC-MS) Analysis

An aliquot of the freshly prepared extract was centrifuged at 9,660 *g* (12,000 rpm) for 10 min. The supernatant of the extract was filtered through a 0.2 μm PTFE membrane for non-targeted metabolite profiling. The profiling analysis was performed with Agilent 6560 Ion Mobility Quadrupole Time-of-Flight (IM-QTOF) coupled with the Agilent 1290 UHPLC (Agilent Technologies, Santa Clara, CA, USA) (24).

The chromatographic separation was carried out through POROSHELL 120 EC-C18 reverse phase chromatographic column (100 × 4.6 mm i.d., 2.7 μm particle size) (Agilent Technologies, Santa Clara, CA, USA) at 40°C. The mobile phase consisted of (A) 0.1% formic acid in water and (B) 0.1% formic acid in acetonitrile. A gradient elution for the metabolite separation was as follows: 5% B (0.0 – 2.0 min), isocratic at 5% B (2.0 – 4.0 min), 5 – 20% B (4.0 – 6.0 min), 20 – 25% B (6.0 – 8.0 min), 25 – 30% B (8.0 – 10.0 min), 30 – 35% B (10.0 – 12.0 min), 35 – 40% B (12.0 – 14.0 min), 40 – 45% B (14.0 – 16.0 min), 45 – 50% B (16.0 – 18.0 min), 50 – 55% B (18.0 – 20.0 min), 55 – 60% B (20.0 – 22.0 min), 60 – 5% B (22.0 – 24.0 min), and isocratic at 5% (24 – 25 min). The flow rate of the eluent through the column were 0.3 ml/min. The injection volume of the extract was 5 μl.

Whereas, the mass spectra data were accomplished with IM-QTOF mass spectrometer (MS). The mass spectra were recorded across the range of m/z between 100 and 1,000. The electrospray ionisation (ESI-MS) acquisition of the metabolites was in positive (ES+) mode. The MS operating conditions were set with a capillary voltage of 4,000 V, nozzle voltage of 500 V, fragmentor voltage of 365 V, the nebulizer pressure (N_2_) was kept at 20 psi, drying gas temperature was maintained at 225°C, drying gas flow was 12 L/min and sheath gas flow was 12 L/min at 400°C. There was a Dual Agilent Jet Stream Technology (Dual AJS ESI) channel in the ESI compartment to ensure the desired mass accuracy of the recorded ions. The technology worked through continuous internal calibration in the compartment with the reference ion solution of protonated purine and protonated hexakis [(1H,1H,3H-tetrafluoropropoxy) phosphazine or HP-921], in which carried the signals at m/z 121.0509 and 922.0098, respectively.

Both the LC system and MS data acquisition were monitored and controlled with Agilent Data Acquisition (version B.06.00) software. The instrument was calibrated and tuned each time before running the LC-MS analysis. Deionized water was used as the background blank.

### Data Processing and Analysis

#### Data Processing

The acquired spectral raw data were subjected to recursive molecular feature extraction (MFE) algorithm through Agilent MassHunter Profinder software (version B.06.00) to extract the reliable features or metabolites. The features were extracted *via* chromatographic deconvolution with minimum 1,000 counts of the peak height to avoid the noise spectral picking. The internal reference ions in the MS system and adduct ions of [M+H]^+^, [M+Na]^+^, and [M+NH_4_]^+^ were considered during the feature extraction process in the recorded mass spectra.

The extracted features were then aligned across all the data with tolerances window of retention time (RT) 0.1 min and the mass of 2.0 mDa. The recursive workflow was employed to perform a targeted feature extraction with reference of the m/z value, mass and RT of each feature that been extracted with MFE algorithm to minimise the appearance of both false positive and negative metabolites. To reduce the signal redundancy, the identical elution profile with different m/z values were merged into a compound group and further handled as a single variable. This eased the deletion of the false-positive features from the blank.

There were no metabolites detected and extracted in one of the samples from Sabah and Pahang, specifically S03 and C02, respectively after recursive MFE algorithm. Hence, the metabolites of only 63 EBN samples were exported as compound exchange format (.cef files) for subsequent analysis and interpretation.

#### Data Pre-treatment and Mining

The metabolite features from data processing (.cef files) were then imported into Agilent Mass Profiler Professional (MPP) software (version 13.1.1) for data pre-treatment and mining before multivariate analysis. Data pre-treatment was carried out across the sample set *via* filtering with minimum 5,000 intensities peak; alignment of RT and mass with a tolerance window of 0.01 min and 2.0 mDa, respectively. Normalisation was done with 75 percentile shift algorithm and the baseline was transformed to the median of all the samples.

The data matrix was based on 63 EBN observations and few thousands of metabolite variables. Since the number of metabolites was greater than the number of observations, data mining was carried out to retain the important metabolites. Stepwise reduction filtering was performed based on the frequency of occurrence and results of Kruskal-Wallis with the multiple testing correction of Benjamini Hochberg False Discovery Rate. Values were considered statistically significant at *p* < 0.05.

#### Metabolite Identification

The identification of the retained metabolites was further done with Agilent MassHunter ID Browser. The software deduced the empirical formula of each metabolite by evaluating its accurate ion mass and isotopic profile. The accuracy of each metabolite with the assigned empirical formula was calculated as a score. The accurate mass and RT (optional) of the metabolite were searched against METLIN database. The tolerance of the compound identity matching was restricted to ±5 ppm and 0.1 min (optional).

Indisputable confirmation of the compound identities was not performed with the use of chemical standards as well as MS/MS fragmentation. Therefore, the identification performed was considered as tentative in this study.

#### Multivariate Analysis

Multivariate analysis was carried out to interpret the large and complex data set through Agilent Mass Profiler Professional (MPP) software (version 13.1.1). The data were logarithmically transformed to lower relatively large differences among the respective metabolite abundances. Un-supervised principle component analysis (PCA) and hierarchical clustering analysis (HCA) were carried out to examine the differences between house EBN through pattern recognition.

HCA was carried out to cluster concurrently on both EBNs and metabolite variables. Pearson's centred correlation and average linkage were used to compute the distance metric and linkage rule for the hierarchical clustering, respectively.

The decision on the number of clusters to retain from HCA was interpreted *via* the hypothesis testing on the significance of the correlation coefficient. The significance of the correlation coefficient had been calculated using *t*-distribution and the significance level of the hypothesis testing was set as 5% (α = 0.05). Venn diagram was used to investigate the metabolites that found among all the house EBNs in Malaysia. To understand the relationship among the house EBN samples from different location in Malaysia, the distance and geographical distribution surrounded the swiftlet houses were studied through Google map.

## Results

### LC-MS Data Acquisition of House EBNs

Non-targeted metabolite profiling was applied to obtain the EBN profiles with most of the possible metabolites to compare for the differences. Based on the visual inspection of the raw total ion chromatograms (TIC), the house EBNs from Malaysia showed minor variation, both from the same and different states (Supplementary Figure 1). The differences were observed between the retention time of 3–5 min and 7–15 min in all the chromatographic patterns which could be molecular fingerprint information for the EBNs.

There was no metabolite found to be significantly different from Kruskal-Wallis statistical test. Approximately 34% of metabolites (1,987 metabolites) were retained from the stepwise reduction filtering for analysis. However, there were only 674 metabolites identified and 669 metabolites with only empirical formula were found among 1,987 metabolites. Therefore, a final amount of 1,343 metabolite variables were subjected for the subsequent multivariate analysis to determine the relationship of the 63 house EBNs from different localities in Malaysia.

### Unsupervised Multivariate Analysis on the Metabolites Profile of EBNs

#### Principle Component Analysis

Unsupervised PCA analysis was performed to determine the variability trends between the house EBNs from all 13 states in Malaysia through the approach of dimensionality reduction in the metabolites profile of EBNs. The PCA score plot for the EBNs is displayed in Supplementary Figure 2. The first two principal components (PC1 and PC2) plot in PCA that accounted the most total variance was only at 34.5 and 11.43%. This cumulative variance of 45.93% in PCA was unable to show the significant variation among the EBNs from different locations as it was <70%. The results suggest that either the EBN samples are more similar than they are different or more variable was required to explain the compositional differences among the EBNs (25).

#### Hierarchical Clustering Analysis

**Figure 2:**
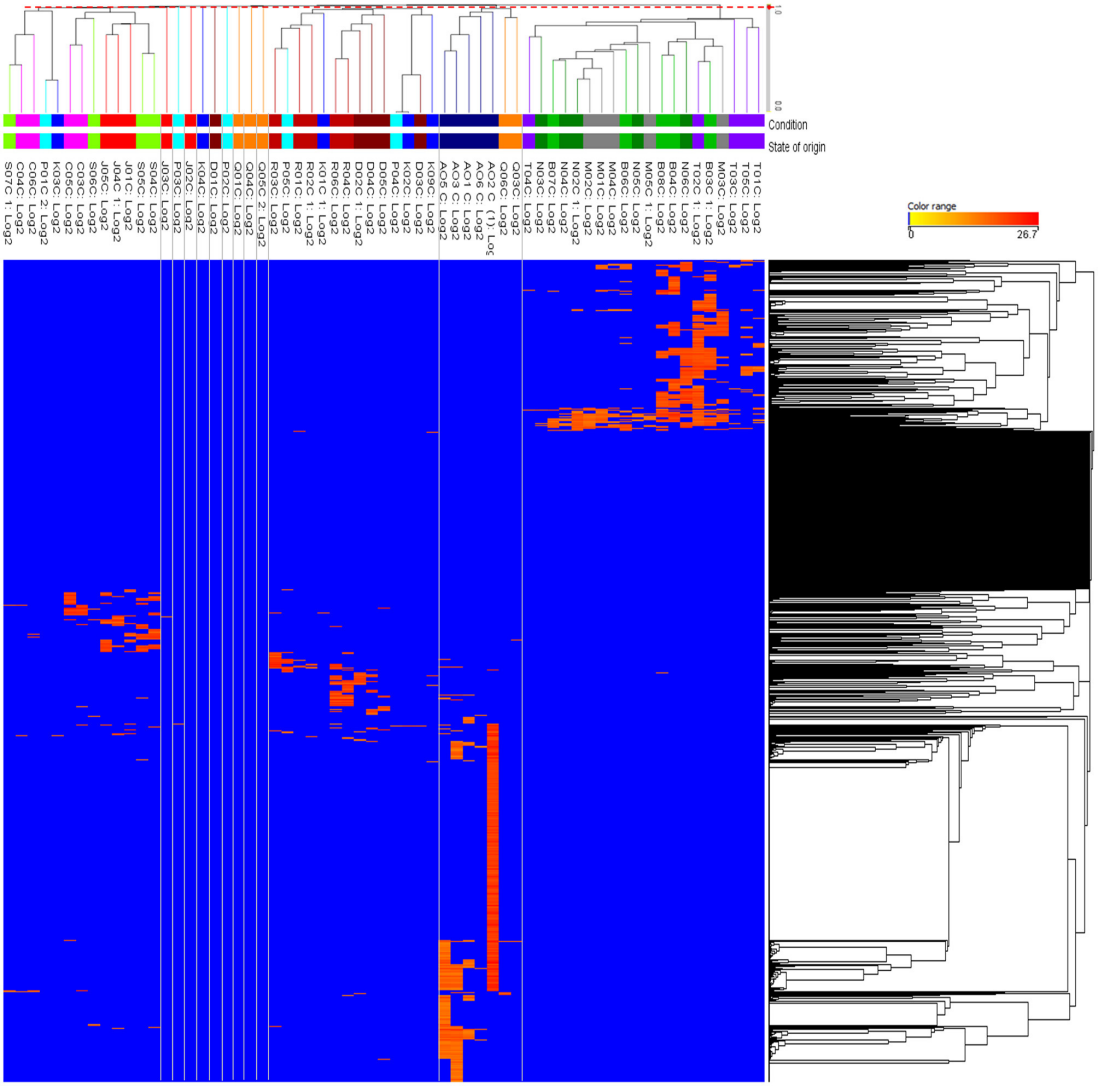
Hierarchical clustering heatmap of the EBNs from 63 different swiftlet houses corresponding to their metabolites. The clustering of the entities was based on the total metabolites of 1,343 across all the EBN samples. The columns represent the individual 63 EBNs and the rows represent the metabolites. The colour range bar indicates the normalised intensity of each metabolite from absent or low concentration (blue) to high concentration (orange). The threshold distance (*d* = 0.9779) in red dotted line which across the dendrogram was determined with hypothesis testing on the significance of correlation coefficient (α = 0.05) to retain 13 clusters for the EBN samples. The clusters are demarcated with the grey lines.

Since PCA was unable to reveal clear variation between the house EBN samples, HCA was performed to organise and group between the metabolites and the 63 EBN samples based on the similarities in the metabolite occurrence pattern. This could reveal the holistic relationship in the complex metabolic data of EBNs and provide an overview of all the house EBN samples. The results are presented on the dendrogram with the heatmap in Figure 2 to show the clustering between house EBNs and the metabolites.

The hypothesis testing on the minimum significance correlation coefficient in the dendrogram showed that there were 13 clusters retained among the 63 EBN samples. However, only four clusters attained meaningful information whereas the remaining nine clusters were comprised of individual samples, which was defined as the outliers. The state represented in each cluster was defined as the occurrence of at least two biological EBN samples. Hence, the four clusters were Cluster 1 with all the biological samples from Selangor, Melaka, Negeri Sembilan, Terengganu; Cluster 2 with the samples from Perak and majority from Sarawak; Cluster 3 which was mostly from Perlis, Kelantan, Kedah, Penang and Cluster 4 included the EBN samples majority from Sabah, Pahang and Johor. The information of the samples that group in each cluster is summarised in Table 1. The retained four clusters are tally with the unique pattern of metabolites distribution in HCA (Figure 2).

**Table 1:** Summary samples list in each of the clusters.

**Cluster**	**Samples**	**States**
1	B03, B04, B06, B07, B08, M01, M02, M03, M04, M05, N02, N03, N04, N05, N06, T01, T02, T03, T04, T05	Selangor, Melaka, Negeri Sembilan, Terengganu
2	A01, A02, A03, A05, A06, Q03, Q06	Perak, Sarawak
3	R01, R02, R03, R04, R06, D02, D03, D04, D05, K01, K02, K09, P04, P05	Perlis, Kelantan, Kedah, Penang
4	S04, S05, S06, S07, C03, C04, C05, C06, J01, J04, J05, K03, P01	Sabah, Pahang, Johor, Kedah, Penang

*The cluster sequence is from right to left according to Figure 2*.

Among the retained clusters, all the five biological EBN samples from eight states were well-defined under a cluster. The eight states included Selangor, Melaka, Negeri Sembilan, Terengganu, Perak, Perlis, Sabah and Pahang. On the other hands, the EBN samples from Kedah and Penang were divided separately into two clusters (Clusters 3 and 4).

### Metabolites Distribution

The number of metabolites that elucidate the four main clusters is shown in Table 2. Out of the total metabolites found in each cluster, there were only 6.9–41.18% of metabolites identified through METLIN database matching and retained from the filtering criteria. The information of all the identified and retained metabolites which elucidated each cluster is detailed in Supplementary Table 2.

**Table 2:** The number of metabolites in EBNs that elucidate each cluster.

**Cluster**	**Localities of Samples**	**Samples quantity**	**Total metabolites**	**Identified metabolites**	Differentiated metabolites^*^	**Uniqueness of metabolites (%)**
1	Selangor, Melaka, Negeri Sembilan, Terengganu	20	261	18	260	99.62
2	Perak, Sarawak	7	455	121	383	84.18
3	Perlis, Kelantan, Kedah, Penang (P04, P05)	14	103	36	49	47.57
4	Johor, Sabah, Pahang, K03, P01	13	102	42	48	47.06

*The identified metabolites were filtered and retained with the criteria of having the score higher than 80, the database matching differences lower than ±5 ppm and any contaminants*.

* *The differentiated metabolites are the metabolites which is not repeated in other clusters. Result obtained from the Venn diagram*.

**Figure 3:**
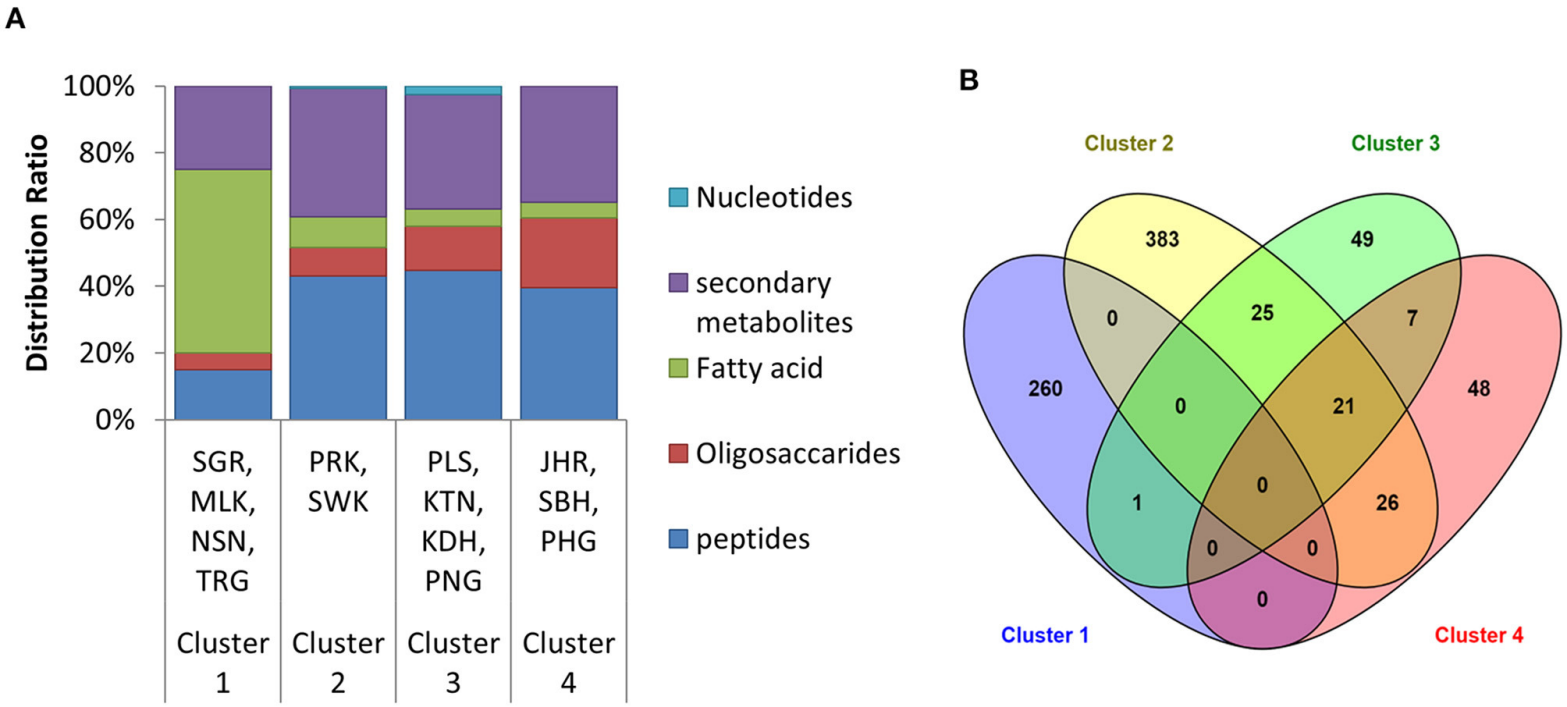
**(A)** The type of metabolites distributed in each of the clusters. The distribution was based on the retained identified metabolites in the EBNs. **(B)** The Venn diagram elucidated the relationship between the four clusters based on the total metabolites distribution (the overall identified metabolites and the unidentified metabolites with molecular formula) in each cluster. SGR, Selangor; MLK, Melaka; NSN, Negeri Sembilan; TRG, Terengganu; PRK, Perak; SWK, Sarawak; PLS, Perlis; KTN, Kelantan; KDH, Kedah; PNG, Penang; JHR, Johor; SBH, Sabah; PHG, Pahang.

Based on retained and identified metabolites, the types of the metabolite distribution were further classified into five groupings, including oligosaccharides, peptides, fatty acids, nucleotides, and secondary metabolites. The classification of the metabolite distribution is illustrated in Figure 3A. The results showed that the distribution of the metabolites was slightly different in the composition ratio in different clustering of house EBNs. Cluster 1 was comprised with the highest composition of fatty acid. Whereas, the content of oligosaccharides in the EBN samples from Cluster 4 was the highest. Although the metabolite distribution was found to be slightly different, the major metabolites which characterised in all the clusters (except Cluster 1) were peptides, followed by secondary metabolites, oligosaccharides and fatty acid. Based on the metabolite distribution in HCA (Figure 2) and the identities of the metabolites (Supplementary Table 2), it was noted that the metabolites which drove each cluster formation have happened in a group instead of individual key metabolite.

#### The Similar Metabolites of EBNs in All the Clusters

Since the results of HCA were based on the similarity in the metabolite profile, it was interesting to know the metabolite that found to be similar either between or among the clusters. Venn diagram was further investigated in this study (Figure 3B). The result showed there was no metabolite found to be similar among all the four retained clusters. However, there were still some similar metabolites with slightly different intensities were found between the clusters, such as between Clusters 2 and 3; Clusters 2 and 4; Clusters 3 and 4 as well as among Clusters 2, 3, and 4. The identities of the metabolites that found to be similar between the clusters are denoted in Supplementary Table 2. The result from the Venn diagram further suggested that the clustering in HCA was not only based on the presence/absence of metabolites but also quantitative differences of metabolites among the samples.

The Venn diagram result displayed in Figure 3B shows that Cluster 1 has no metabolites found similarly with the other three clusters. However, one unidentified metabolite with the formula of C_39_H_56_N_11_O_2_S was found to be similar to Cluster 3. This disclosed the uniqueness of the EBNs from the state of Selangor, Melaka, N. Sembilan and Terengganu with 99.62% (Table 2), as compared with the other location of Malaysia. On the other hand, Cluster 2 was found to has high number of similar metabolites with other clusters, in which 25 and 26 metabolites with Clusters 3 and 4, respectively (Figure 3B). Although Cluster 2 shared abundance metabolites with other clusters, the distinguishable metabolites in Cluster 2 remained its uniqueness with 84.18% (Table 2). Whereas, Clusters 3 and 4 displayed lesser uniqueness with their grouping as compared with Clusters 1 and 2. The metabolite classes that found to be similar between/among Clusters 2, 3, and/or 4 were mostly comprised either peptides or secondary metabolites or both.

### Geographical Distribution

**Figure 4:**
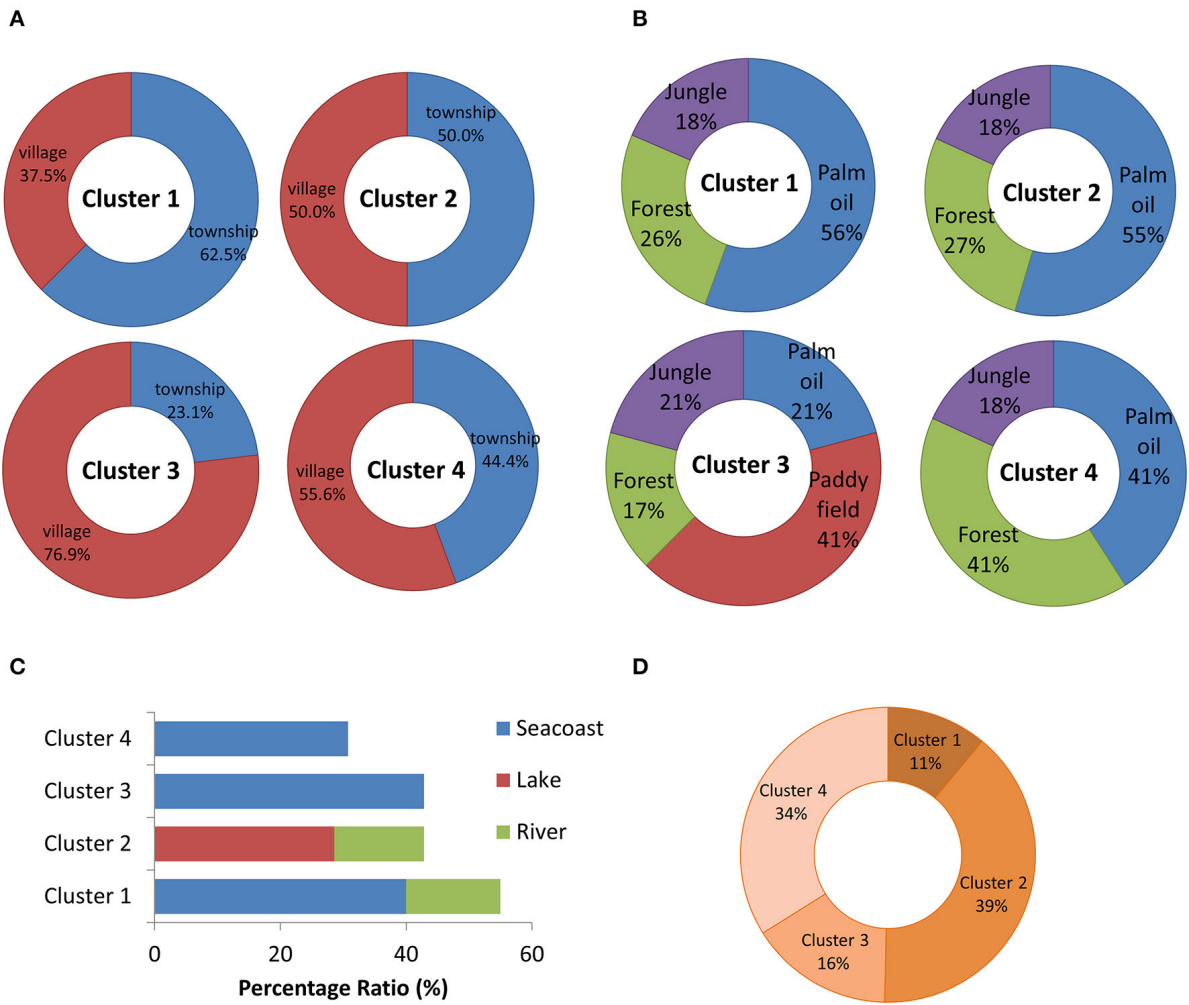
The distribution ratio of the geographical factors with the classification based on **(A)** development area, **(B)** food source availability, **(C)** water source, and **(D)** mountains/hilly area. The distribution was based on the sample localities in each cluster. The samples localities in Cluster 1 is Selangor, Melaka, N. Sembilan, Terengganu; Cluster 2 is Perak, Sarawak; Cluster 3 is Perlis, Kelantan, Kedah, Penang and Cluster 4 is Johor, Sabah and Pahang.

Geographical distribution surrounded the sampling swiftlet houses were investigated by categorised into four segments, which included the status of the development area, the food sources availability, water sources and the presence of mountains (Figure 4). The status of the development area and the food sources availability nearby the swiftlet houses in Cluster 1 (Selangor, Melaka, N. Sembilan, Terengganu) and Cluster 2 (Perak, Sarawak) were most likely similar, where both of the swiftlet houses were located mostly in the township area with a large proportion of oil palm field availability of 56 and 55%, respectively. However, the different distribution of mountains and water sources near to the swiftlet houses further differentiate both of Clusters 1 and 2. In which, the distributions of mountain were higher in Cluster 2. Whereas, the houses in Cluster 1 were mostly located near to the seacoast and the houses in Cluster 2 were mostly located nearby the lakeside.

Although the swiftlet houses in Cluster 3 (Perlis, Kelantan, Kedah, and Penang) and Cluster 4 (Johor, Sabah, and Pahang) were mostly located in the village near to the sea coast, the distribution of the plantation fields and mountains further lead to the uniqueness of both clusters. The uniqueness of Cluster 3 swiftlet houses was mostly located on or near to the paddy fields with low availability of mountains. Whereas, the swiftlet houses in Cluster 4 were close to the high distribution of mountain area with equal distribution of oil palm and forest. In short, the geographical distribution that represents in each cluster were unique with different availability and distribution of plantation field, water source, mountains and the degree of urbanisation.

## Discussion

### Multivariate Analysis and Grouping of EBNs

In this study, the differences of the house EBNs throughout Malaysia was unable to be grouped with PCA. The results obtained were similar to the finding of Chua et al. (20), where the PCA unable to resolve the differences between the EBN samples despite the sample size is large. We speculated that the non-parametric (not normally distributed) dataset in this study failed to achieve the assumption criteria of the analysis, which further led to poor grouping of EBN in PCA (26). On the other hands, the HCA combined with correlation coefficient showed that there were some differences in the house EBNs in Malaysia, mainly with four clusters formation. The result of EBN clustering is supported with the finding of Seow et al. (4), which the grouping was not according to the common classification system of the regions based on the states in Malaysia. For example, Perak and Sarawak were grouped in Cluster 2 despite these states are separated geographically by the South China Sea. To further investigate the factor that contributes to the result, distance and geographical distribution were studied.

### Influence of Distance Between the Swiftlet Houses

The distance between the swiftlet houses was examined according to the clustering result of EBNs. It was found that despite the distance between the swiftlet houses is very close, with <5 km distance in the area, EBNs were grouped separately in different clusters. For example, the EBNs of K03 and K04 from Kedah; EBNs of P01, P02, and P05 from Penang; EBNs of Q03, Q04, Q05, and Q06 from Sarawak; and EBNs of J03 and J05 from Johor.

Meanwhile, some of the EBNs were grouped as the same cluster although the swiftlet houses were located further, either within or between the states. For example, the EBNs from Perak and Sarawak in Cluster 2 which geographically separated by the South China Sea. Besides, one of the swiftlet houses was grouped under Cluster 1 despite it located further with 62.6 km from other houses in Selangor. The similar phenomena also occurred to the swiftlet houses in Perak (Cluster 2), Kedah (Cluster 3), Pahang and Sabah (Cluster 4) with the range of 30–162 km distance.

These findings could be the inference that the distance between the different swiftlet houses was not the factor that affects the differences of the EBNs. The finding was further supported by Lee et al. (27) in which the tea samples were different in their metabolites although the samples were originated from the area that was close to each other. Since the differences of the house EBNs were not due to the distance between the swiftlet houses, geographical distribution around the swiftlet houses was further investigated in this study.

### Relationship Between Geographical Distribution and Clustering of EBNs

The investigation of geographical distribution in each cluster further revealed the differences of EBNs. However, some exceptional cases were found where the EBNs obtained from the locations with similar geographical distribution were grouped into different clusters. For example, the EBNs of J03 and J05 from Johor; K03 and K04 from Kedah; D01 and D02 from Kelantan; P01, P02, and P05 from Penang; as well as the EBNs of Q05 and Q06 from Sarawak. Meanwhile, some EBNs from different geographical distribution were grouped under a similar cluster. For instances, EBNs of M04 and M05 from Melaka and T03 from Terengganu were grouped in Cluster 1; Q03 and Q06 from Sarawak were grouped in Cluster 2 together with Perak; as well as P04 and P05 from Penang were grouped in Cluster 3.

Since the diet is one of the most important resource axes along which ecologically separated (28). The diet of insectivorous swiftlet according to the geographical distribution on the landscape was reviewed to provide an explanation to the four clusters formation and the exceptional cases as described above.

#### Types of Plantation Fields With Diverse Insect Orders and Species

Swiftlets that produced EBNs were primarily feeding with insect orders of *Hymenoptera* (ants, bees, wasps) and *Diptera* (two-winged flies) in almost all the plantation field types (29–31). However, swiftlet does include some other combination of insect orders as their diet in different fields, despite the major *Hymenoptera* and *Diptera*. The preference of insect orders may similar or varied between the field types as reviewed in Table 3.

**Table 3:** The preference of swiftlet on the insect orders and species present in different plantation fields.

**Plantation fields**	**Rice cultivation**	**Oil palm**	**Forest**
References	Salmah et al. (31)	Syed-Ab-Rahman et al. (30)	Lourie and Tompkins (29)
Amount of insect orders in the field	6–9	12	> 6
Insect orders	Diptera	Diptera	Diptera
	Hymenoptera	Hymenoptera	Hymenoptera
	Coleoptera	Hemiptera	Coleoptera
	Neuroptera	Homoptera	Homoptera
	Isoptera	Isoptera	
	Lepidoptera		
Insect species	Ceratopagonidae (biting midges)	Ceratopagonidae	Brachycera
(Diptera order)	Culicidae (mosquito)	Culicidae	
	Chironomidae (biting midges)	Tephritidae (fruit fly)	
	Tipulidae (crane fly)	Asilidae (robber fly)	
		Psychodidae (moth fly)	

Although swiftlet depends on *Hymenoptera* and *Diptera* majorly, each of the insect orders is nevertheless comprised of megadiverse insect species with approximately or more than 150,000 species (32, 33). The presence of insect species is not random and highly depends on the abiotic factors, the presence of predators, parasites and competitors in a location (34). Therefore, the insect species present depend on the types of plantation field, even though they are from a similar insect order. The presence of insect species from the same insect order in different plantation fields is shown in Table 3.

Thus, the option of swiftlet with different insect orders and the presence of diverse insect species allow the production of EBNs to be grouped according to the distribution of the plantation field types such as oil palm in Cluster 1 and 2, rice cultivation in Cluster 3, and forest which characterised Cluster 4.

#### Habitats of Insects

Although Clusters 1 and 2 were grouped into different cluster, it was found that the distribution of plantation field types in both clusters was almost similar. This result may explain with the observation of Syed-Ab-Rahman et al. (30), where swiftlet tends to forage for different insect species at different locations despite the similar types of plantation. For example, swiftlet forage for *Asilidae* and *Ceratopogonidae* species (*Diptera* order) in the oil palm field at Perak; whereas the swiftlet in the same field at Kelantan feed on *Tephritidae* and *Culicidae* (*Diptera* order). Moreover, the insect species in the same plantation field are found to be varied in different sites of the field such as margin, interior and beneficial flowering plants area (35). Hence, the differences in the distribution of insect species at different sites and locations of the plantation field may contribute to the grouping of Clusters 1 and 2 as well as the exceptional cases, in which EBNs from similar geographical distribution were grouped into different clusters.

#### Status of Developed Area

Insect of *Hymenoptera* order with slightly larger in size is found predominantly in the undeveloped forest. However, the abundance of *Hymenoptera* reduced in rural followed by urban areas. Whereas, the abundance of *Diptera* insects is vice versa of *Hymenoptera* in the forest to the township (29, 36). Thus, swiftlet in the township area tends to forage more on *Diptera*, while the swiftlet in the village prefers *Hymenoptera* insects. This further explained the clustering of EBNs as the natural distribution of insects depends on the development of a location.

The differences in size between the common preference *Hymenoptera* and *Diptera* might affect the behaviour of the swiftlet's diet. Swiftlet might increase its consumption if the size of insects is smaller. Consequently, the ingested insect diversity may increase with the number of insects in the diet of swiftlet and contribute to the differences in EBN. However, not much research is found regarding the relationship between the size, amount, and diversity of insects in the diet of swiftlet.

#### Plant Phenology and Water Source

The abundances and diversity of insect species in the paddy field were not consistent throughout the year. This phenomenon was noticed to be closely related to the rice growth phenology and level of water usage in rice cultivation (31). Since the nature requirement on the damp or aquatic habitats is highly valued for the growth of larvae which are susceptible to drying, the availability of larvae in the field is highly contributed to the distribution of adult insects (32). Larvae from different insect species favour different damp terrestrial and aquatic habitats, thus the different water sources that characterised the clusters (Cluster 1 and 2) will attract the inhabitant of different insect species.

Besides, the minerals composition of insects is varied in the habitat with different types of water sources. Such differences had contributed to the mineral levels in the EBN (19, 21). This further explained the importance and the effect of water sources on the insect and subsequently contributed to the EBNs. Meanwhile, the different rice cultivation phases will attract different species of insects by providing food and habitat. Therefore, the distribution of insects depends on the types of water sources and the growth stage of crops could lead to the differences in the production of EBNs by insectivorous swiftlet.

#### Emergence Temporal of Insects

Insects tend to emerge in a swarm at different temporal to avoid the competitors and predators (37). For example, *Coleoptera* and *Lepidoptera* appeared mostly in crepuscular and nocturnal temporal periods, *Hymenoptera* has a broader temporal from matutinal to nocturnal; while *Diptera* is diurnal insects. The emergence temporal of insects contributed to the diet preference of swiftlet. Since swiftlet is active in diurnal foraging, *Hymenoptera* and *Diptera* were mostly captured. Hence, the differences in EBN were also affected by the insect species captured according to the factor of temporal.

#### Foraging Habits of Swiftlet

Swiftlet is not particularly selective on their diet composition (insect diversity) but reacts with the food availability (insect density). The higher abundances of the insect will contribute to the most common dietary items of swiftlet (28, 29, 36, 38, 39). Therefore, it was found that swiftlet does not consume all the insect orders distributed in any landscapes (Table 3).

By integrating the results and the explanation abovementioned, the availability and diversity of insects will vary depends on geography, seasonality, plant phenology as well as temporal impact. Consequently, all these influences have increased the degree of variability in the preferred insects in the diet of swiftlet. Such influences were able to observe from the foraging behaviour of swiftlet with food availability. Swiftlet will change their foraging manoeuvres and position in an airspace according to temporal variation (40). It was also found that the home range and core range were slightly varied in swiftlets in an area (41). Therefore, the uniqueness of geographical distribution in each cluster contributes to the distribution of insect species, which subsequently contributed to the differences in metabolite distribution in EBNs.

### Metabolites Distribution

The average nutritional composition in insects are mostly from protein with 37–61.4%, followed by fat and carbohydrates (42). The average composition of insects is found slightly homogeneous to the metabolite distribution in Clusters 2, 3, and 4. The metabolite profiling in this study and the average nutritional composition of insects is found to be similar to the proximate and elemental analysis of EBNs, in which protein is the highest content followed by carbohydrates and fats (1, 15). The results have shown the influences of insects in the diet of swiftlet on the production of EBNs.

However, the average nutritional composition of insects as above-mentioned does not fully represent all the insects. The components will differently depend on the species and the growth stage of insects (42, 43). Since insect distribution was geographically dependent, the self-feeding behaviour of swiftlet in the natural environment will increase the variation in the metabolite distribution during the EBN production. Hence, it was observed that the metabolite distribution pattern was slightly different in each cluster, especially Clusters 1 and 2 with an almost similar distribution of township and oil palm area. In short, the metabolites distribution of EBNs was affected by the preferred diet of swiftlet depending on the insect availability which fundamentally linkable to the geographical distribution. However, the relationship among the geographical distribution, ingestion of the type of insects by swiftlet and the metabolites profile of EBN should be studied in-depth in the future.

Furthermore, secondary metabolites in this study were found to be comparable with peptides in all the clusters. The ingested secondary metabolites in plants by insects might contribute to the swiftlet and the production of EBN. Therefore, apart from the natural synthesis of secondary metabolites in swiftlet, the presence of forests and jungles were viewed as important external sources to the abundances of secondary metabolites in EBN. This claim further showed that the dynamics ecosystem is closely influenced by primary productivity to related population dynamics (44, 45). However, more studies in the future are required to prove this claim.

### The Relation Between Recuperative Effects and Metabolite of EBN

This study revealed the metabolite profile of EBNs, which may provide possible explanations for the recuperative effects of EBN. The potential secondary metabolite with the identity of 6-hydroxymelatonin (6-OHM) in Clusters 2, 3, and 4 suggested to help in antioxidant and neuroprotective benefits. The 6-OHM is an intermediate metabolite of melatonin through photo-degradation, which dominant in the nucleus and mitochondria of hepatocytes (46–48). This metabolite is effective in reducing oxidative damages than its parent melatonin. The presence of phenol moiety on 6-OHM can reduce lipid peroxidation by directly scavenge the reactive oxygen species (ROS), such as peroxyl radicals, hydroxyl radicals and superoxide anions. Besides, 6-OHM exhibits antioxidative effect by indirectly sequester metals induced oxidation and enhancing the DNA repairing system (46, 47, 49, 50). All these mechanisms in antioxidant properties of 6-OHM allow it to protect the cells from DNA damage and the neuron cells from the toxicity effects induced by the oxidative stress. Therefore, the presence of 6-OHM in EBN may be responsible for its antioxidant effects and might be able to cure oxidative stress-associated diseases such as neurodegeneration (Parkinson's and Alzheimer's disease), inflammation, diabetes and arthritis.

The fatty acid of 1, 25-Dihydroxy-24-oxo-16-ene-vitamin D3 [lα, 25(OH)_2_-24-oxo-16-ene-D3] found in Cluster 1 is a stable intermediary metabolite of 1α,25(OH)_2_-16-ene-D3, the analogue to vitamin D3. The metabolite of lα, 25(OH)_2_-24-oxo-16-ene-D3 was found with its ability to inhibit the proliferation of human chronic myeloid leukaemia cells (51). Besides, this fatty acid is proved to prevent the experimental autoimmune encephalitis (EAE) by suppressing the autoimmune system without inducing hypercalcemia (52). EAE is a local inflammation disease by the autoimmune system that contributes to axonal and myelin damage and further lead to neurodegeneration (53, 54). Therefore, the 1α,25(OH)_2_-24-oxo-16-ene-D3 in this study may be related to the anti-inflammatory and neuroprotective effects of EBN, which may help to treat EAE-like multiple sclerosis in the future.

The metabolites identified in this study can provide insight into the possible mediated functions of EBN, further studies on structure elucidation and quantitation with standard compounds for metabolite validation are required in the future. In addition, the mediated function of the suggested metabolites should be carried out in the future *via* functional assays for further confirmation.

## Conclusion

The house EBNs from different localities in Malaysia exhibited the differences by forming four main clusters through hierarchical clustering analysis (HCA) combined with the hypothesis testing on the correlation coefficient. The clusters that displayed the most distinctiveness were Cluster 1 (Selangor, Melaka, Negeri Sembilan, and Terengganu) and Cluster 2 (Perak and Sarawak). The metabolites drove the differences of EBN occurred in a group instead of a single major metabolite. The metabolites that characterised Cluster 1 were fatty acids, while the rest of the clusters were peptides and secondary metabolites. The model proposed by HCA mostly coincides with the metabolite profiles of house EBNs from all the 13 states of Malaysia. Therefore, HCA combined with correlation coefficient can be used to group the EBNs from different localities in Malaysia. However, further validation of the model is still required in the future by using supervised partial least squares-discriminatory analysis (PLS-DA).

Taken together in this study, the differences in terms of metabolite distribution in house EBNs in Malaysia were not due to the distance between the located swiftlet houses. Instead, the most probable factor in influencing the variation in the house EBNs was the geographical factors. Swiftlets have to adapt their foraging area and behaviour following the distribution of insects that closely dependent on geographical distribution. Such behaviour has further caused minor variation in the preferred diet of swiftlet. Consequently, the nutritional composition of the consumed insects is different by the swiftlet and caused the metabolite distribution of EBNs to be different.

The metabolite profile of EBNs in this study unfolded the major metabolites of EBNs, this includes peptides, followed by secondary metabolites and fatty acids. The secondary metabolites were found as important metabolites in EBNs which worth further study. Besides, the metabolites found in this study have partly revealed the possible explanations of their bioactivities of EBNs. However, structure elucidation, quantification and functional assays of the interested metabolites of EBN should be carried out in the future.

## Data Availability Statement

The original contributions presented in the study are included in the article/Supplementary Material, further inquiries can be directed to the corresponding author/s.

## Author Contributions

Y-ML conceived and designed the study and reviewed the manuscript. S-RT carried out experiments, analysed/interpreted data, and prepared the manuscript. T-HL took responsibility in sample collection and performed the extraction on the samples. S-KC, T-HL, and Y-ML provided input and advice to the study. All authors contributed to the article and approved the submitted version.

## Conflict of Interest

The authors declare that the research was conducted in the absence of any commercial or financial relationships that could be construed as a potential conflict of interest.

## References

[1] Ma F Liu D. Sketch of the edible bird's nest and its important bioactivities. Food Res Int. (2012) 48:559–67. 10.1016/j.foodres.2012.06.001

[2] Marcone MF. Characterization of the edible bird's nest the “Caviar of the East.” Food Res Int. (2005) 38:1125–34. 10.1016/j.foodres.2005.02.008

[3] Wong RS. Edible bird's nest: food or medicine? Chin J Integr Med. (2013) 19:643–9. 10.1007/s11655-013-1563-y 23975128

[4] Seow E-K Ibrahim B Muhammad SA Lee LH Cheng L-H. Differentiation between house and cave edible bird's nests by chemometric analysis of amino acid composition data. LWT Food Sci Technol. (2016) 65:428–35. 10.1016/j.lwt.2015.08.047

[5] Guo CT Takahashi T Bukawa W Takahashi N Yagi H Kato K. Edible bird's nest extract inhibits influenza virus infection. Antiviral Res. (2006) 70:140–6. 10.1016/j.antiviral.2006.02.005 16581142 PMC7114130

[6] Aswir AR Wan Nazaimoon WM. Effect of edible bird's nest on caco-2 cell proliferation. J Food Technol. (2010) 8:126–30. 10.3923/jftech.2010.126.130

[7] Abidin FZ Chua KH Ng SL Ramli ESM Lee TH Ghafar NA. Effects of edible bird's nest (EBN) on cultured rabbit corneal keratocytes. BMC Compl Altern Med. (2011) 11:94. 10.1186/1472-6882-11-94 21992551 PMC3213154

[8] Aswir A Wan Nazaimoon W. Effect of edible bird's nest on cell proliferation and tumor necrosis factor-alpha (TNF-α) release *in vitro*. Int Food Res J. (2011) 18:1123–7.

[9] Matsukawa N Matsumoto M Bukawa W Chiji H Nakayama K Hara H. Improvement of bone strength and dermal thickness due to dietary edible bird's nest extract in ovariectomized rats. Biosci Biotechnol Biochem. (2011) 75:590–2. 10.1271/bbb.100705 21389609

[10] Roh KB Lee J Kim YS Park J Kim JH Lee J. Mechanisms of edible bird's nest extract-induced proliferation of human adipose-derived stem cells. Evid Based Compl Alternat Med. (2012) 2012:797520. 10.1155/2012/797520 22110547 PMC3206510

[11] Chua KH Lee TH Nagandran K Md Yahaya NH Lee CT Tjih ET. Edible Bird's nest extract as a chondro-protective agent for human chondrocytes isolated from osteoarthritic knee: *in vitro* study. BMC Compl Altern Med. (2013) 13:19. 10.1186/1472-6882-13-19 23339380 PMC3558384

[12] Yida Z Imam MU Ismail M. *In vitro* bioaccessibility and antioxidant properties of edible bird's nest following simulated human gastro-intestinal digestion. BMC Compl Altern Med. (2014) 14:468. 10.1186/1472-6882-14-468 25475744 PMC4289220

[13] Hou Z Imam MU Ismail M Azmi NH Ismail N Ideris A. Lactoferrin and ovotransferrin contribute toward antioxidative effects of Edible Bird's Nest against hydrogen peroxide-induced oxidative stress in human SH-SY5Y cells. Biosci Biotechnol Biochem. (2015) 79:1570–8. 10.1080/09168451.2015.1050989 26057702

[14] Hou Z Imam MU Ismail M Ismail N Zhang Y Ideris A. Effects of edible bird's nest on hippocampal and cortical neurodegeneration in ovariectomized rats. Food Funct. (2015) 6:1701–11. 10.1039/C5FO00226E 25920003

[15] Lee TH Wani WA Tan ETT Adnan N Ling YL Aziz RA. Investigations into the physicochemical, biochemical and antibacterial properties of Edible Bird's Nest. J Chem Pharm Res. (2015) 7:228–47.

[16] Lau ASM Melville DS. International trade in swiftlet nests with special reference to Hong Kong. In: Gray J editor, Species in Danger. Cambridge: TRAFFIC International (1994).

[17] Looi QH Omar AR. Swiftlets and Edible Bird's Nest industry in Asia. Pertanika J Scholar Res Rev. (2016) 2:32–48.

[18] Huang X Li Z Xiaobo Z Shi J Tahir HE Xu Y. Geographical origin discrimination of edible bird's nests using smart handheld device based on colorimetric sensor array. J Food Measur Character. (2019) 14:514–26. 10.1007/s11694-019-00251-z

[19] Saengkrajang W Matan N Matan N. Nutritional composition of the farmed edible bird's nest (*Collocalia fuciphaga*) in Thailand. J Food Compos Anal. (2013) 31:41–5. 10.1016/j.jfca.2013.05.001

[20] Chua YG Bloodworth BC Leong LP Li SF. Metabolite profiling of edible bird's nest using gas chromatography/mass spectrometry and liquid chromatography/mass spectrometry. Rapid Commun Mass Spectrom. (2014) 28:1387–400. 10.1002/rcm.6914 24797951

[21] Seow EK Ibrahim B Muhammad S Lee LH Lalung J Cheng L. Discrimination between cave and house-farmed Edible Bird's Nest based on major mineral profiles. Pertanika J Trop Agric Sci. (2016) 39:181–95.

[22] Quek MC Chin NL Yusof YA Law CL Tan SW. Characterization of edible bird's nest of different production, species and geographical origins using nutritional composition, physicochemical properties and antioxidant activities. Food Res Int. (2018) 109:35–43. 10.1016/j.foodres.2018.03.078 29803459

[23] Vaclavik L Lacina O Hajslova J Zweigenbaum J. The use of high performance liquid chromatography-quadrupole time-of-flight mass spectrometry coupled to advanced data mining and chemometric tools for discrimination and classification of red wines according to their variety. Anal Chim Acta. (2011) 685:45–51. 10.1016/j.aca.2010.11.018 21168550

[24] Tong SR Lee TH Cheong SK Lim YM. Untargeted metabolite profiling on the water-soluble metabolites of edible bird's nest through liquid chromatography-mass spectrometry. Vet World. (2020) 13:304–16. 10.14202/vetworld.2020.304-316 32255973 PMC7096308

[25] Hughey CA McMinn CM Phung J. Beeromics: from quality control to identification of differentially expressed compounds in beer. Metabolomics. (2015) 12:5. 10.1007/s11306-015-0885-5

[26] Grootveld M. Chapter 1: introduction to the applications of chemometric techniques in “omics” research: common pitfalls, misconceptions and “rights and wrongs.” In: Grootveld M editor. Metabolic Profiling: Disease and Xenobiotics. Cambridge: The Royal Society of Chemistry (2015). p. 1–34. 10.1039/9781849735162-00001

[27] Lee JE Lee BJ Chung JO Kim HN Kim EH Jung S. Metabolomic unveiling of a diverse range of green tea (*Camellia sinensis*) metabolites dependent on geography. Food Chem. (2015) 174:452–9. 10.1016/j.foodchem.2014.11.086 25529705

[28] Asokan S Ali AMS Manikannan R. Diet of three insectivorous birds in Nagapattinam District, Tamil Nadu, India – a preliminary study. J Threat Taxa. (2009) 1:327–30. 10.11609/JoTT.o2145.327-30

[29] Lourie SA Tompkins DM. The diets of Malaysian swiftlets. Int J Avian Sci. (2000) 142:596–602. 10.1111/j.1474-919X.2000.tb04459.x

[30] Syed-Ab-Rahman SFMNH Burhanuddin M. Diversity of the insects in the diet of edible nest swiftlets in oil palm plantations. J Biodiv Environ Sci. (2016) 8:39–48.

[31] Salmah MR Siregar AZ Hassan A Nasution Z. Dynamics of aquatic organisms in a rice field ecosystem: effects of seasons and cultivation phases on abundance and predator-prey interactions. Trop Ecol. (2017) 58:177–91.

[32] Merritt RW Courtney GW Keiper JB. Chapter 76 - diptera: (flies, mosquitoes, midges, gnats). In: Resh VH Cardé RT editors, Encyclopedia of Insects. 2nd ed. San Diego: Academic Press (2009). p. 284–97. 10.1016/B978-0-12-374144-8.00085-0

[33] Quicke DLJ. Chapter 127 - hymenoptera: ants, bees, wasps. In: Resh VH Cardé RT editors, Encyclopedia of Insects. 2nd ed. San Diego: Academic Press (2009). p. 473–84. 10.1016/B978-0-12-374144-8.00136-3

[34] Zwick P. Chapter 23 - biogeographical patterns. In: Resh VH Cardé RT editors, Encyclopedia of Insects. 2nd ed. San Diego: Academic Press (2009). p. 82–91. 10.1016/B978-0-12-374144-8.00032-1

[35] Khairiyah MHS Elfira SE Hanysyam NM Nurdiana S Norashirene MJ Faezah P. Entomofaunal diversity of diptera at FELDA besout 6 oil palm plantation. IERI Procedia. (2013) 5:45–50. 10.1016/j.ieri.2013.11.068

[36] Looi QH Ideris A Abu Bakar MZ Omar AR. Morphology comparison of swiftlet species from natural and man-made habitats in Malaysia. Sains Malays. (2015) 44:497–502. 10.17576/jsm-2015-4404-03

[37] Ting JS Abd Rahman NA Ng YF Yaakop S Akbar Z. Insect diversity and abundance during the crepuscular and nocturnal temporal periods in the Kota Gelanggi Limestone Complex, Pahang, Malaysia. Serangga. (2016) 21:97–113.

[38] Lim CK Cranbrook E. Swiftlets of Borneo: Builders of Edible Nests. Sabah: Natural History Publication (Borneo). (2002).

[39] Nituda CJP Nuneza OM. Diet Composition of two species of swiftlets from caves of Northern Mindanao, Philippines. Bull Env Pharmacol Life Sci. (2016) 5:48–52.

[40] Manchi SS Sankaran R. Foraging habits and habitat use by edible-nest and glossy swiftlets in the Andaman Islands, India. Wilson J Ornithol. (2010) 122:259–72. 10.1676/09-144.1

[41] Burhanuddin M Hafidzi MN. Ranging behaviour of edible nest swiftlet (*Aerodramus sp*.) in Kuala Langat district, Selangor, Malaysia. Malaysian Appl Biol. (2017) 46:59–66.

[42] Ahmad H Ong SQ Tan EH. The diet for edible-nest swiftlets: nutritional composition and cost of life stages of *Megaselia scalaris* Loew (Diptera: Phoridae) bred on 3 commercial breeding materials. Int J Insect Sci. (2019) 11:1–5. 10.1177/1179543318823533 30675104 PMC6330733

[43] Rumpold BA Schluter OK. Nutritional composition and safety aspects of edible insects. Mol Nutr Food Res. (2013) 57:802–23. 10.1002/mnfr.201200735 23471778

[44] Dyer LA. Multidimensional diversity associated with plants: a view from a plant-insect interaction ecologist. Am J Bot. (2018) 105:1439–42. 10.1002/ajb2.1147 30151878

[45] Beran F Kollner TG Gershenzon J Tholl D. Chemical convergence between plants and insects: biosynthetic origins and functions of common secondary metabolites. New Phytol. (2019) 223:52–67. 10.1111/nph.15718 30707438

[46] Maharaj DS Glass BD Daya S. Melatonin: new places in therapy. Biosci Rep. (2007) 27:299–320. 10.1007/s10540-007-9052-1 17828452

[47] Alvarez-Diduk R Galano A Tan DX Reiter RJ. N-acetylserotonin and 6-hydroxymelatonin against oxidative stress: implications for the overall protection exerted by melatonin. J Phys Chem B. (2015) 119:8535–43. 10.1021/acs.jpcb.5b04920 26079042

[48] Gagnière J Bonnet M. Chapter 15 - molecular mechanism underlying the actions of antioxidant molecules in digestive disorders. In: Gracia-Sancho J SalvadóJ editors, Gastrointestinal Tissue. London, UK: Academic Press (2017). p. 197–216. 10.1016/B978-0-12-805377-5.00014-X

[49] Perez-Gonzalez A Castaneda-Arriaga R Alvarez-Idaboy JR Reiter RJ Galano A. Melatonin and its metabolites as chemical agents capable of directly repairing oxidized DNA. J Pineal Res. (2019) 66:e12539. 10.1111/jpi.12539 30417425

[50] Wang J Wang X He Y Jia L Yang CS Reiter RJ. Antioxidant and pro-oxidant activities of melatonin in the presence of copper and polyphenols *in vitro* and *in vivo*. Cells. (2019) 8:80903. 10.3390/cells8080903 31443259 PMC6721667

[51] Siu-Caldera ML Clark JW Santos-Moore A Peleg S Liu YY Uskoković MR. 1α,25-dihydroxy-24-oxo-16-ene vitamin D3, a metabolite of a synthetic vitamin D3 analog, 1α,25-dihydroxy-16-ene vitamin D3, is equipotent to its parent in modulating growth and differentiation of human leukemic cells. J Steroid Biochem Mol Biol. (1996) 59:405–12. 10.1016/S0960-0760(96)00134-3 9010346

[52] Lemire JM Archer DC Reddy GS. 1,25-Dihydroxy-24-OXO-16ene-vitamin D3, a renal metabolite of the vitamin D analog 1,25-dihydroxy-16ene-vitamin D3, exerts immunosuppressive activity equal to its parent without causing hypercalcemia *in vivo*. Endocrinology. (1994) 135:2818–21. 10.1210/endo.135.6.7988477 7988477

[53] Constantinescu CS Farooqi N O'Brien K Gran B. Experimental autoimmune encephalomyelitis (EAE) as a model for multiple sclerosis (MS). Br J Pharmacol. (2011) 164:1079–106. 10.1111/j.1476-5381.2011.01302.x 21371012 PMC3229753

[54] Robinson AP Harp CT Noronha A Miller SD. The experimental autoimmune encephalomyelitis (EAE) model of MS: utility for understanding disease pathophysiology and treatment. Handb Clin Neurol. (2014) 122:173–89. 10.1016/B978-0-444-52001-2.00008-X 24507518 PMC3981554

