# Supplementary material for: Geographical Factor Influences the Metabolite Distribution of House Edible Bird's Nests in Malaysia
Source: Front Nutr. 2021 Jun 28;8:658634. doi: 10.3389/fnut.2021.658634 (PMC8273228; doi:10.3389/fnut.2021.658634)
Supplement: Supplementary file 1 [file Data_Sheet_1.docx]

Supplementary Material


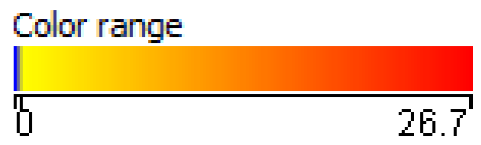
**Supplementary Table 1.** Gazetteer: the coordinates of localities of the different swiftlet houses.

| **States** | **Samples** | **Location** | **GPS** | | **States** | **Samples** | **Location** | **GPS** | |
| --- | --- | --- | --- | --- | --- | --- | --- | --- | --- |
| Perak | A01 | Kampar | 4.312 N | 101.152 E | Negeri Sembilan | N02 | Port Dickson | 2.512 N | 101.827 E |
|  | A02 | Mambang Diawan | 4.280 N | 101.146 E |  | N03 | Seremban | 2.633 N | 101.967 E |
|  | A03 | Bidor | 4.117 N | 101.294 E |  | N04 | Seremban | 2.696 N | 101.946 E |
|  | A05 | Malim Nawar | 4.370 N | 101.122 E |  | N05 | Seremban | 2.701 N | 101.964 E |
|  | A06 | Chemor | 4.700 N | 101.084 E |  | N06 | Seremban | 2.716 N | 101.947 E |
| Selangor | B03 | Batang Berjuntai | 3.378 N | 101.406 E | Penang | P01 | Georgetown | 5.417 N | 100.326 E |
|  | B04 | Kuala Langat | 2.808 N | 101.496 E |  | P02 | Georgetown | 5.419 N | 100.331 E |
|  | B06 | Kuala Langat | 2.771 N | 101.468 E |  | P03 | Seberang Prai | 5.201 N | 100.496 E |
|  | B07 | Kuala Langat | 2.665 N | 101.558 E |  | P04 | Nibong Tebal | 5.173 N | 100.476 E |
|  | B08 | Kuala Langat | 2.824 N | 101.536 E |  | P05 | Georgetown | 5.406 N | 100.327 E |
| Pahang | C02 | Bera | 3.271 N | 102.454 E | Perlis | R01 | Arau | 6.328 N | 100.196 E |
|  | C03 | Bentong | 3.522 N | 101.910 E |  | R02 | Arau | 6.434 N | 100.302 E |
|  | C04 | Raub | 3.794 N | 101.857 E |  | R03 | Arau | 6.426 N | 100.273 E |
|  | C05 | Jerantut | 3.937 N | 102.362 E |  | R04 | Kangar | 6.334 N | 100.165 E |
|  | C06 | Kuala Lipis | 4.184 N | 102.054 E |  | R06 | Kangar | 6.384 N | 100.152 E |

**Supplementary Table 1. (continued)**

| **States** | **Samples** | **Location** | | **GPS** | | **States** | **Samples** | | **Location** | | **GPS** | | |  |  |
| --- | --- | --- | --- | --- | --- | --- | --- | --- | --- | --- | --- | --- | --- | --- | --- |
| Kelantan | D01 | Kota Bharu | 6.117 N | | 102.278 E | Terengganu | T01 | Marang | | 5.143 N | | 103.087 E |  |  |  |
|  | D02 | Kota Bharu | 6.117 N | | 102.278 E |  | T02 | Marang | | 5.028 N | | 103.294 E |  |  |  |
|  | D03 | Kuala Krai | 5.531 N | | 102.202 E |  | T03 | Kuala Terengganu | | 5.335 N | | 103.139 E |  |  |  |
|  | D04 | Tanah Merah | 5.809 N | | 102.147 E |  | T04 | Dungun | | 4.744 N | | 103.420 E |  |  |  |
|  | D05 | Tanah Merah | 5.809 N | | 102.147 E |  | T05 | Dungun | | 4.755 N | | 103.416 E |  |  |  |
| Johor | J01 | Batu Pahat | 1.853 N | | 102.926 E | Sarawak | Q01 | Sibu | | 2.299 N | | 111.698 E |  |  |  |
|  | J02 | Batu Pahat | 1.848 N | | 102.859 E |  | Q03 | Sibu | | 2.263 N | | 111.844 E |  |  |  |
|  | J03 | Tangkak | 2.268 N | | 102.534 E |  | Q04 | Sibu | | 2.247 N | | 111.838 E |  |  |  |
|  | J04 | Muar | 2.013 N | | 102.696 E |  | Q05 | Sibu | | 2.329 N | | 111.957 E |  |  |  |
|  | J05 | Tangkak | 2.267 N | | 102.540 E |  | Q06 | Sibu | | 2.356 N | | 111.952 E |  |  |  |
| Kedah | K01 | Kota Kuala Muda | 5.587 N | | 100.376 E | Sabah | S03 | Beaufort | | 5.345 N | | 115.745 E |  |  |  |
|  | K02 | Kota Kuala Muda | 5.647 N | | 100.354 E |  | S04 | Membakut | | 5.526 N | | 115.697 E |  |  |  |
|  | K03 | Langkawi | 6.334 N | | 99.871 E |  | S05 | Pulau jambongan | | 6.757 N | | 117.471 E |  |  |  |
|  | K04 | Langkawi | 6.334 N | | 99.871 E |  | S06 | Beaufort | | 5.274 N | | 115.684 E |  |  |  |
|  | K09 | Kubang Pasu | 6.373 N | | 100.437 E |  | S07 | Membakut | | 5.997 N | | 116.132 E |  |  |  |
| Melaka | M01 | Unknown | - | | |  |  |  |  |  | |  |  |  |  |
|  | M02 | Unknown | - | | |  |  |  |  |  | |  |  |  |  |
|  | M03 | Bandar Baru Jasin | 2.313 N | | 102.432 E |  |  |  | |  | |  |  | |  |
|  | M04 | Alor Gajah | 2.352 N | | 102.109 E |  |  |  | |  | |  |  | |  |
|  | M05 | Central of Melaka | 2.263 N | | 102.216 E |  |  |  | |  | |  |  | | |

**
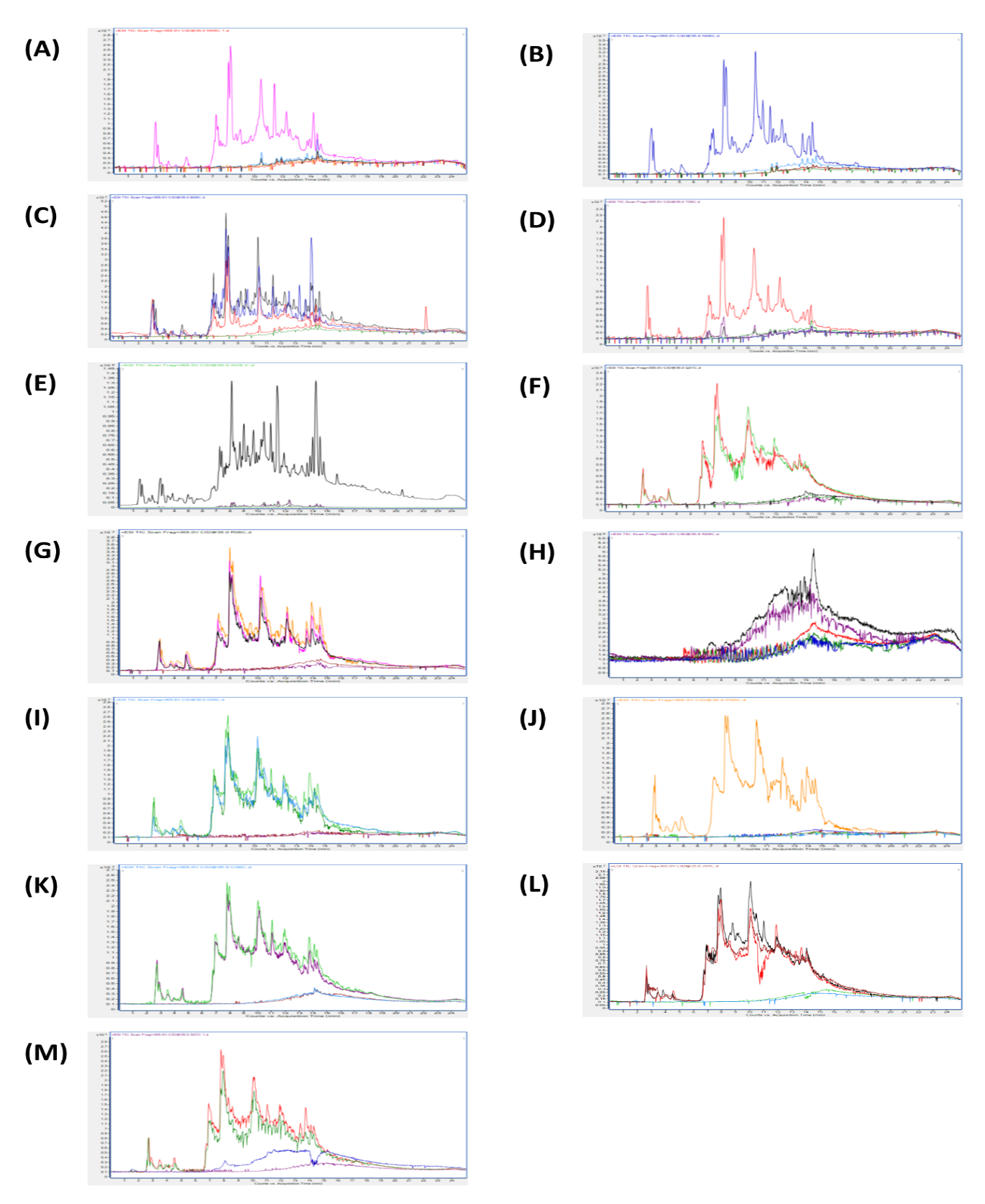
Supplementary Figure 1.** The total ion chromatograms of EBN samples from the states of **(A)** Melaka, **(B)** Negeri Sembilan, **(C)** Selangor, **(D)** Terengganu, **(E)** Perak, **(F)** Sarawak, **(G)** Perlis, **(H)** Kedah, **(I)** Kelantan, **(J)** Penang, **(K)** Pahang, **(L)** Johor and **(M)** Sabah. Each of the chromatograms represents 5 biological replicate samples from different swiftlet houses. The LCMS chromatograms were obtained from ESI+ mode.


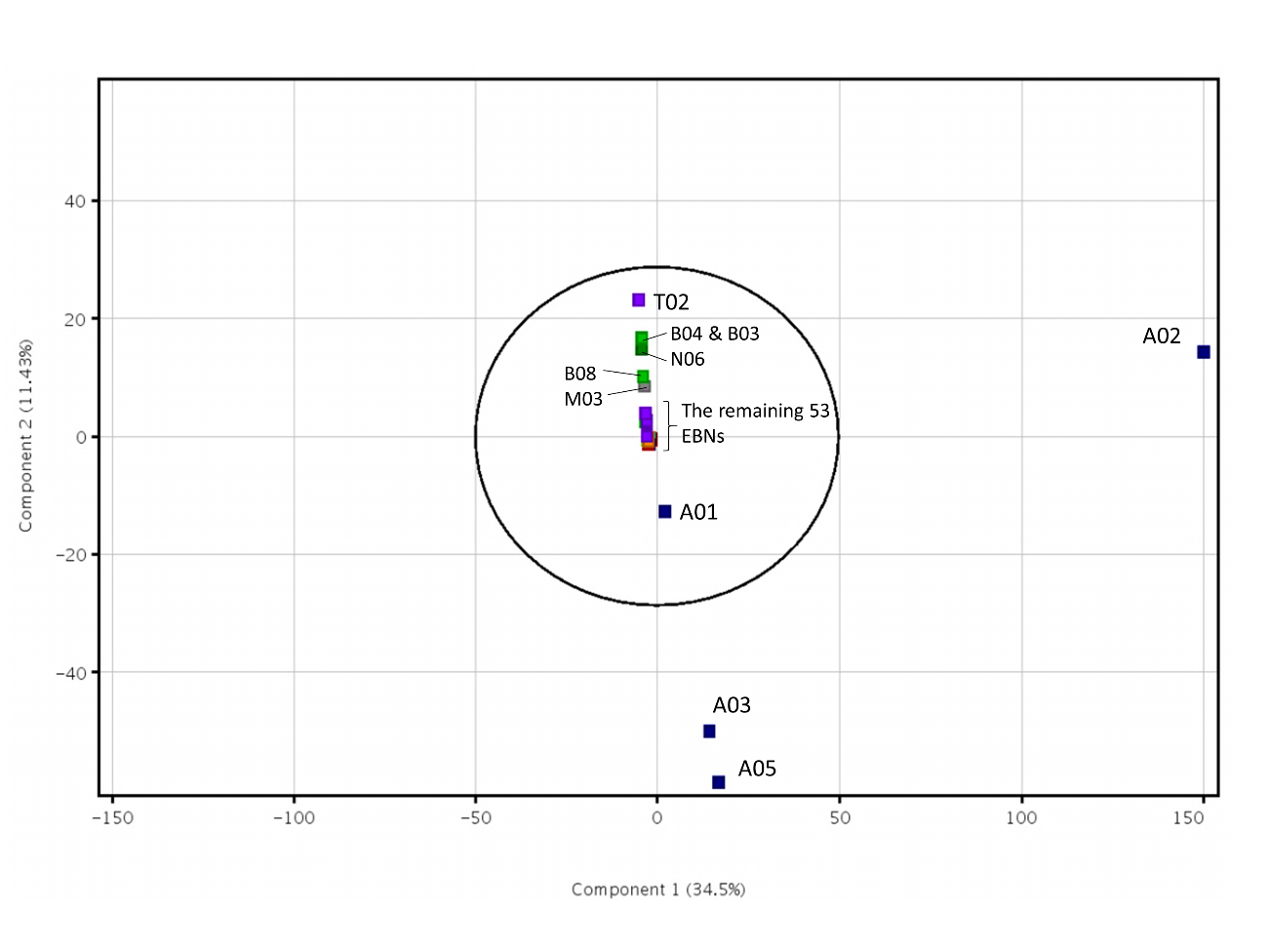


**Supplementary Figure 2.** 2D PCA score plot constructed based on the metabolite profiles to classify the EBNs from the different swiftlet houses in Malaysia. Johor ( ), Kedah ( ), Kelantan ( ), Melaka ( ), Negeri Sembilan ( ), Pahang ( ), Penang ( ), Perak ( ), Perlis ( ), Sarawak ( ), Perlis ( ), Selangor ( ) and Terengganu ( ).


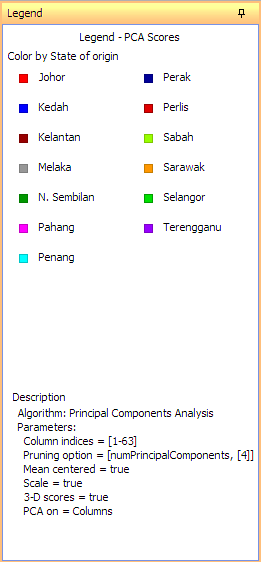

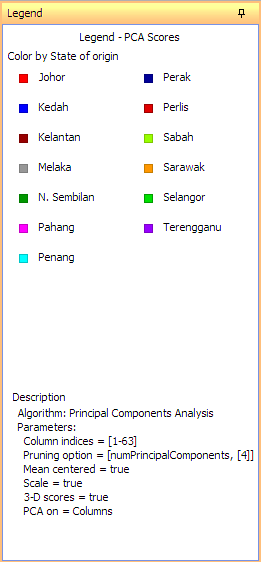

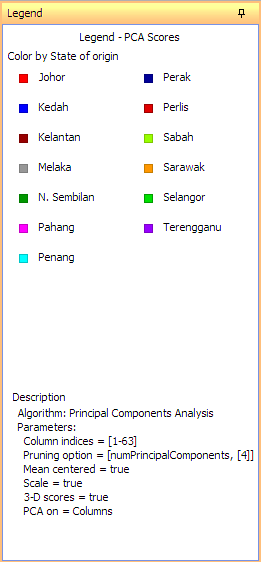

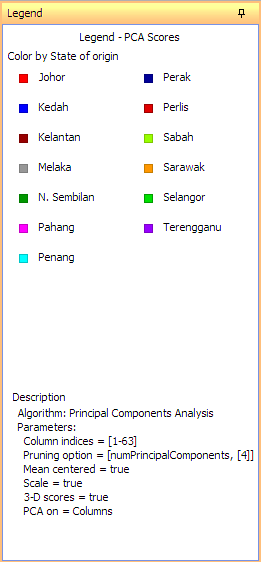

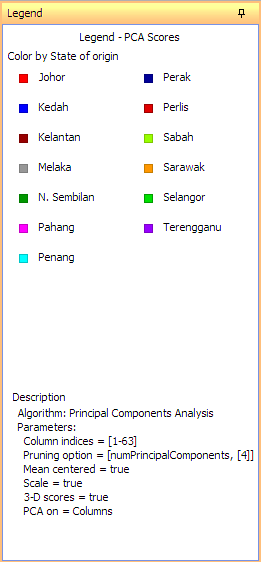

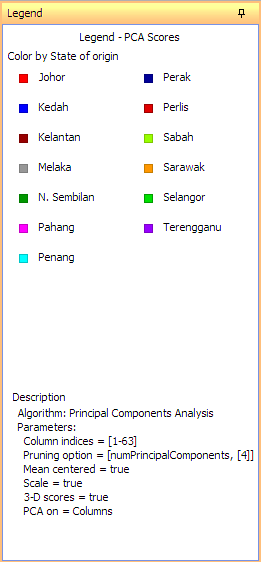

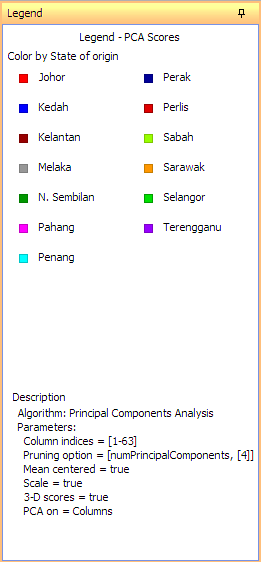

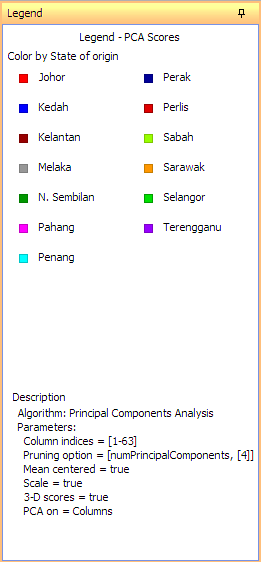

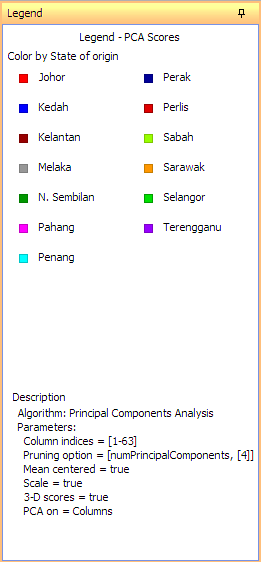

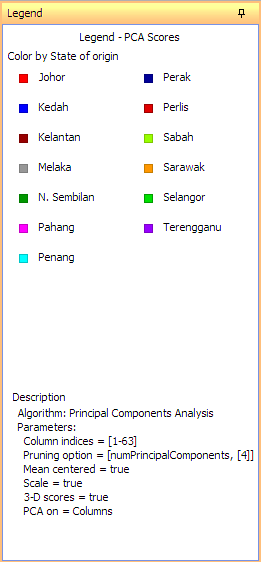

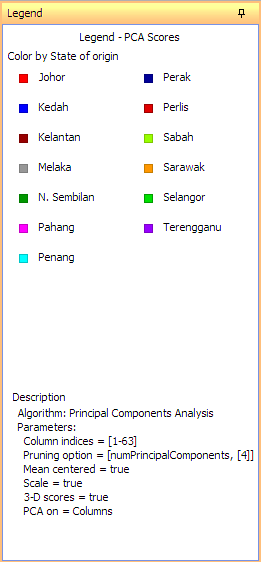

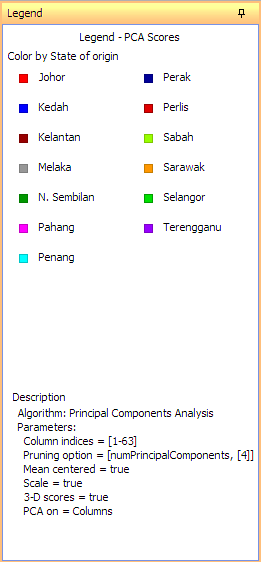

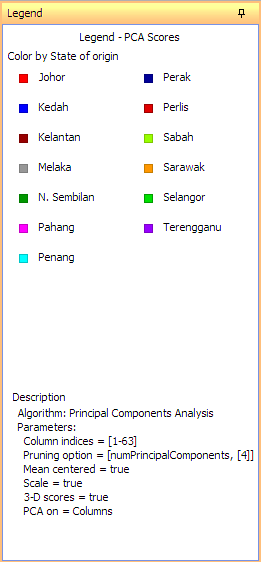


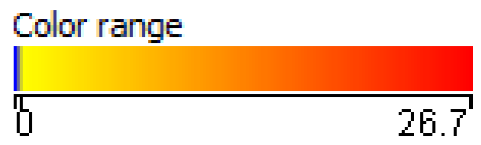
**Supplementary Table 2.** Information of the retained identified metabolites in each cluster.

| **No.** | **RT (min)** | **Ion** | **Mass** | **m/z** | **Molecular formula** | **Score** | **DB differences (ppm)** | **Tentative identified metabolites** | **Metabolites Classification** |
| --- | --- | --- | --- | --- | --- | --- | --- | --- | --- |
| **Cluster 1 (Selangor, Melaka, Negeri Sembilan and Terengganu)** | | | | | | | | | |
| 1 | 14.20 | (M+H)^+^ | 204.1505 | 205.1581 | C_14_H_20_O | 92.73 | 2.10 | (6R,7S)-6,7-Epoxy-1,3-tetradecadiyne | Secondary metabolite |
| 2 | 8.31 | (M+H)^+^ | 206.1671 | 207.1738 | C_14_H_22_O | 80.86 | 3.62 | 1-(2,6,6-Trimethyl-2-cyclohexen-1-yl)-1-penten-3-one / 6-methylionone | Secondary metabolite |
| 3 | 14.46 | (M+NH_4_)^+^ | 790.4636 | 808.4966 | C_40_H_71_O_13_P | 96.97 | -0.41 | 1-(9Z,12Z-heptadecadienoyl)-2-(9Z-tetradecenoyl)-glycero-3-phospho-(1'-myo-inositol) / PI(17:2(9Z,12Z)/14:1(9Z)) | Fatty acid |
| 4 | 13.92 | (M+H)^+^ | 428.2918 | 429.2992 | C_27_H_40_O_4_ | 93.15 | 1.74 | 1,25-Dihydroxy-24-oxo-16-ene-vitamin D3 | Fatty acid |
| 5 | 10.83 | (M+H)^+^ | 900.6484 | 901.6557 | C_57_H_88_O_8_ | 91.89 | -0.24 | 1,2-di-(9Z,12Z,15Z-octadecatrienoyl)-3-(8-(2E,4Z-decadienoyloxy)-5,6-octadienoyl)-sn-glycerol | Fatty acid |
| 6 | 11.90 | (M+H)^+^ | 208.2189 | 209.2262 | C_15_H_28_ | 84.41 | 1.03 | 1,6-Pentadecadiene | Fatty acid |
| 7 | 10.93 | (M+H)^+^ | 214.1940 | 215.2014 | C_13_H_26_O_2_ | 93.60 | -3.47 | 10,10-dimethyl-undecanoic acid | Fatty acid |
| 8 | 8.01 | (M+H)^+^ | 204.1719 | 205.1791 | C_11_H_24_O_3_ | 82.81 | 3.24 | 2-Octylglycerol | Fatty acid |
| 9 | 12.26 | (M+H)^+^ | 166.0814 | 167.0884 | C_10_H_14_S | 89.50 | 1.59 | 3-(4-Methyl-3-pentenyl)thiophene | Secondary metabolite |
| 10 | 3.11 | (M+NH_4_)^+^ | 315.1689 | 333.2028 | C_16_H_21_N_5_O_2_ | 92.90 | 1.85 | Alizapride | Secondary metabolite |
| 11 | 7.26 | (M+H)^+^ | 705.4432 | 706.4507 | C_39_H_63_NO_10_ | 86.19 | 2.78 | beta1-Chaconine | Secondary metabolite |
| 12 | 12.26 | (M+H)^+^ | 178.0837 | 179.0910 | C_7_H_14_O_5_ | 85.14 | 2.41 | beta-D-Digitalopyranose / Digitalose | Oligosaccaride |

**Supplementary Table 2. (continued)**

| **No.** | **RT (min)** | **Ion** | **Mass** | **m/z** | **Molecular formula** | **Score** | **DB differences (ppm)** | **Tentative identified metabolites** | **Metabolites Classification** |
| --- | --- | --- | --- | --- | --- | --- | --- | --- | --- |
| 13 | 10.93 | (M+NH_4_)^+^ | 276.2204 | 294.2542 | C_17_H_28_N_2_O | 97.63 | -0.87 | Etidocaine | Peptide |
| 14 | 8.31 | (M+H)^+^ | 417.2885 | 418.2958 | C_25_H_39_NO_4_ | 92.79 | -1.53 | Latanoprost ethyl amide | Peptide |
| 15 | 14.21 | (M+NH_4_)^+^ | 377.2569 | 395.2930 | C_22_H_35_NO_4_ | 91.46 | -0.82 | N-(12S-hydroxy-5Z,8Z,10E,14Z-eicosatetraenoyl)-glycine / 12-HETE-Gly | Fatty acid |
| 16 | 8.45 | (M+H)^+^ | 447.3384 | 448.3455 | C_24_H_49_NO_4_S | 90.92 | -0.44 | N-docosanoyl taurine | Fatty acid |
| 17 | 7.30 | (M+NH_4_)^+^ | 403.3097 | 421.3435 | C_25_H_41_NO_3_ | 91.79 | -2.51 | N-palmitoyl phenylalanine | Fatty acid |
| 18 | 7.61 | (M+H)^+^ | 213.2099 | 214.2172 | C_13_H_27_NO | 81.99 | -2.82 | Tridecanamide | Fatty acid |
| **Cluster 2 (Perak and Sarawak)** | | | | | | | | | |
| 1 | 20.43 | (M+H)^+^ | 248.1527 | 249.1600 | C_14_H_20_N_2_O_2_ | 99.47 | -0.91 | (2S)-N-(3-hydroxy-2,6-dimethylphenyl)piperidine-2-carboxamide | Secondary metabolite |
| 2 | 11.19 | (M+NH_4_)^+^ | 150.0316 | 168.0652 | C_8_H_6_O_3_ | 95.39 | 0.58 | (E)-8-Hydroxy-2-octene-4,6-diynoic acid | Fatty acid |
| 3 | 10.35 | (M+NH_4_)^+^ | 436.2685 | 454.3028 | C_21_H_40_O_9_ | 90.36 | -2.96 | (R)-1-O-[b-D-Apiofuranosyl-(1-2)-b-D-glucopyranoside]-1,3-octanediol | Fatty acid |
| 4 | 14.51 | (M+H)^+^ | 156.0538 | 157.0611 | C_6_H_8_N_2_O_3_ | 84.94 | -1.96 | (S)-3-(Imidazol-5-yl)lactate **^d^** | Oligosaccharide |
| 5 | 3.86 | (M+H)^+^ | 129.0900 | 130.0973 | C_5_H_11_N_3_O | 99.47 | 1.40 | (S)-Piperazine-2-carboxamide **^d^** | Oligosaccharide |
| 6 | 20.43 | (M+H)^+^ | 212.1528 | 213.1601 | C_11_H_20_N_2_O_2_ | 99.45 | -1.55 | 1,4'-Bipiperidine-1'-carboxylic acid | Fatty acid |
| 7 | 8.27 | (M+H)^+^ | 365.1311 | 366.1384 | C_14_H_23_NO_10_ | 96.61 | 2.96 | 2-(acetylamino)-1,5-anhydro-2-deoxy-4-O-b-D-galactopyranosyl-D-arabino-Hex-1-enitol **^b^** | Oligosaccharide |

**Supplementary Table 2. (continued)**

| **No.** | **RT (min)** | **Ion** | **Mass** | **m/z** | **Molecular formula** | **Score** | **DB differences (ppm)** | **Tentative identified metabolites** | **Metabolites Classification** |
| --- | --- | --- | --- | --- | --- | --- | --- | --- | --- |
| 8 | 10.47 | (M+H)^+^ | 230.1064 | 231.1138 | C_13_H_14_N_2_O_2_ | 81.49 | -3.60 | 2,4-Dihydroxytacrine | Secondary metabolite |
| 9 | 12.28 | (M+H)^+^ | 291.0948 | 292.1021 | C_11_H_17_NO_8_ | 98.41 | 2.11 | 2,7-Anhydro-alpha-N-acetylneuraminic acid **^d^** | Oligosaccharide |
| 10 | 12.28 | (M+H)^+^ | 214.0478 | 215.0551 | C_9_H_10_O_6_ | 86.64 | -0.26 | 2-Hydroxy-6-oxonona-2,4-diene-1,9-dioate | Oligosaccharide |
| 11 | 11.46 | (M+H)^+^ | 190.1114 | 191.1186 | C_11_H_14_N_2_O | 84.39 | -3.91 | 2-Methyl-5-hydroxytryptamine **^b^** | Secondary metabolite |
| 12 | 7.15 | (M+H)^+^ | 190.1104 | 191.1176 | C_11_H_14_N_2_O | 86.83 | 1.32 | 3-(2-(Methylamino)ethyl)-1H-indol-5-ol | Secondary metabolite |
| 13 | 7.29 | (M+H)^+^ | 155.0694 | 156.0767 | C_6_H_9_N_3_O_2_ | 85.72 | 0.74 | 3-(Pyrazol-1-yl)-L-alanine | Peptide |
| 14 | 7.35 | (M+NH_4_)^+^ | 118.0430 | 136.0768 | C_8_H_6_O | 80.64 | -9.37 | 3,5,7-Octatriyn-1-ol **^d^** | Fatty acid |
| 15 | 12.76 | (M+H)^+^ | 186.1369 | 187.1442 | C_9_H_18_N_2_O_2_ | 85.46 | -0.68 | 3-[(3-Methylbutyl)nitrosoamino]-2-butanone | Oligosaccharide |
| 16 | 8.70 | (M+H)^+^ | 115.0634 | 116.0713 | C_5_H_9_NO_2_ | 84.72 | -0.60 | 3-Acetamidopropanal **^d^** | Oligosaccharide |
| 17 | 11.20 | (M+H)^+^ | 166.0268 | 167.0341 | C_8_H_6_O_4_ | 87.16 | -1.15 | 3-Formylsalicylic acid **^b^** | Secondary metabolite |
| 18 | 12.29 | (M+H)^+^ | 196.0371 | 197.0443 | C_9_H_8_O_5_ | 98.86 | 0.44 | 3-Methoxy-4,5-methylenedioxybenzoic acid | Secondary metabolite |
| 19 | 14.50 | (M+NH_4_)^+^ | 159.0688 | 177.1027 | C_10_H_9_NO | 85.31 | -2.61 | 3-Methyl-quinolin-2-ol | Secondary metabolite |
| 20 | 9.40 | (M+H)^+^ | 480.2548 | 481.2621 | C_22_H_40_O_11_ | 88.94 | 4.70 | 3-O-(alpha-L-rhamnopyranosyl-(1-2)-alpha-L-rhamnopyranosyl)-3-hydroxydecanoic acid | Fatty acid |
| 21 | 8.29 | (M+H)^+^ | 139.0742 | 140.0814 | C_6_H_9_N_3_O | 93.21 | 2.64 | 4-Amino-5-hydroxymethyl-2-methylpyrimidine **^a^** | Nucleotides |
| 22 | 11.42 | (M+H)^+^ | 446.2319 | 447.2395 | C_25_H_34_O_7_ | 92.53 | -3.33 | 4-epi-clavulone III | Fatty acid |
| 23 | 7.10 | (M+NH_4_)^+^ | 166.0372 | 184.0711 | C_7_H_6_N_2_O_3_ | 85.42 | 3.63 | 4-Hydroxy-3-nitrosobenzamide | Secondary metabolite |

**Supplementary Table 2. (continued)**

| **No.** | **RT (min)** | **Ion** | **Mass** | **m/z** | **Molecular formula** | **Score** | **DB differences (ppm)** | **Tentative identified metabolites** | **Metabolites Classification** |
| --- | --- | --- | --- | --- | --- | --- | --- | --- | --- |
| 24 | 10.27 | (M+H)^+^ | 135.0687 | 136.0760 | C_8_H_9_NO | 87.29 | -2.33 | 5-(2-Furanyl)-3,4-dihydro-2H-pyrrole **^b^** | Secondary metabolite |
| 25 | 10.94 | (M+H)^+^ | 275.1641 | 276.1714 | C_15_H_21_N_3_O_2_ | 93.30 | -2.73 | 5-Hydroxyprimaquine | Secondary metabolite |
| 26 | 10.70 | (M+H)^+^ | 218.1428 | 219.1500 | C_13_H_18_N_2_O | 96.46 | -3.89 | 5-Methoxydimethyltryptamine **^d^** | Secondary metabolite |
| 27 | 14.50 | (M+H)^+^ | 248.1167 | 249.1241 | C_13_H_16_N_2_O_3_ | 95.78 | -2.30 | 6-Hydroxymelatonin **^d^** | Secondary metabolite |
| 28 | 14.29 | (M+H)^+^ | 143.0742 | 144.0814 | C_10_H_9_N | 84.90 | -4.66 | 6-Methylquinoline | Secondary metabolite |
| 29 | 9.00 | (M+NH_4_)^+^ | 363.1228 | 381.1571 | C_20_H_17_N_3_O_4_ | 87.77 | -2.41 | 9-Aminocamptothecin | Secondary metabolite |
| 30 | 7.15 | (M+H)^+^ | 392.2172 | 393.2228 | C_18_H_28_N_6_O_4_ | 92.13 | 4.08 | Ala Phe Arg **^b^** | Peptide |
| 31 | 9.38 | (M+Na)^+^ | 399.1788 | 422.1680 | C_21_H_25_N_3_O_5_ | 98.85 | 1.58 | Ala Phe Tyr | Peptide |
| 32 | 13.97 | (M+H)^+^ | 388.2115 | 389.2190 | C_20_H_28_N_4_O_4_ | 96.38 | -1.24 | Ala Trp Leu **^a^** | Peptide |
| 33 | 13.16 | (M+H)^+^ | 200.0804 | 201.0877 | C_8_H_12_N_2_O_4_ | 93.74 | -3.68 | Alanylclavam | Secondary metabolite |
| 34 | 10.18 | (M+H)^+^ | 186.1008 | 187.1082 | C_8_H_14_N_2_O_3_ | 85.10 | -2.15 | Alanyl-Proline **^d^** | Peptide |
| 35 | 10.93 | (M+H)^+^ | 232.1583 | 233.1656 | C_14_H_20_N_2_O | 97.19 | -3.31 | Albine **^a^** | Secondary metabolite |
| 36 | 3.56 | (M+H)^+^ | 552.3133 | 553.3207 | C_22_H_44_N_6_O_10_ | 95.25 | -2.49 | Arbekacin | Secondary metabolite |
| 37 | 8.11 | (M+NH_4_)^+^ | 141.0793 | 159.1131 | C_7_H_11_NO_2_ | 86.99 | -2.01 | Arecaidine **^d^** | Secondary metabolite |
| 38 | 3.19 | (M+H)^+^ | 385.2078 | 386.2152 | C_15_H_27_N_7_O_5_ | 93.28 | -1.16 | Arg Asn Pro | Peptide |
| 39 | 12.27 | (M+H)^+^ | 386.1903 | 387.1975 | C_15_H_26_N_6_O_6_ | 81.59 | 2.78 | Arg Pro Asp | Peptide |
| 40 | 7.90 | (M+H)^+^ | 523.2542 | 524.2613 | C_26_H_33_N_7_O_5_ | 97.90 | 0.32 | Arg Trp Tyr | Peptide |
| 41 | 2.82 | (M+H)^+^ | 289.1380 | 290.1452 | C_10_H_19_N_5_O_5_ | 83.36 | 1.98 | Arginyl-Aspartate **^b^** | Peptide |
| 42 | 8.70 | (M+H)^+^ | 321.1804 | 322.1875 | C_15_H_23_N_5_O_3_ | 95.15 | -0.96 | Arginyl-Phenylalanine | Peptide |
| 43 | 12.28 | (M+H)^+^ | 271.1656 | 272.1729 | C_11_H_21_N_5_O_3_ | 81.98 | -4.37 | Arginyl-Proline **^d^** | Peptide |
| 44 | 14.51 | (M+H)^+^ | 376.1221 | 377.1293 | C_13_H_20_N_4_O_9_ | 95.04 | 2.53 | Asn Asp Glu | Peptide |

**Supplementary Table 2. (continued)**

| **No.** | **RT (min)** | **Ion** | **Mass** | **m/z** | **Molecular formula** | **Score** | **DB differences (ppm)** | **Tentative identified metabolites** | **Metabolites Classification** |
| --- | --- | --- | --- | --- | --- | --- | --- | --- | --- |
| 45 | 8.03 | (M+H)^+^ | 300.1436 | 301.1510 | C_12_H_20_N_4_O_5_ | 83.33 | -0.83 | Asn Pro Ala | Peptide |
| 46 | 10.51 | (M+NH_4_)^+^ | 378.1904 | 396.2245 | C_18_H_26_N_4_O_5_ | 82.10 | -0.30 | Asn Val Phe | Peptide |
| 47 | 14.30 | (M+H)^+^ | 486.2248 | 487.2322 | C_27_H_34_O_8_ | 96.37 | 1.16 | Austalide I | Secondary metabolite |
| 48 | 14.21 | (M+H)^+^ | 260.1529 | 261.1604 | C_15_H_20_N_2_O_2_ | 95.46 | -1.80 | Baptifoline | Secondary metabolite |
| 49 | 7.16 | (M+H)^+^ | 111.0798 | 112.0870 | C_5_H_9_N_3_ | 87.14 | -1.03 | Betazole **^d^** | Secondary metabolite |
| 50 | 12.30 | (M+H)^+^ | 273.0859 | 274.0930 | C_12_H_19_NO_2_S_2_ | 82.65 | -0.61 | Brugine **^b^** | Secondary metabolite |
| 51 | 13.56 | (M+NH_4_)^+^ | 484.3053 | 502.3392 | C_27_H_40_N_4_O_4_ | 99.47 | -0.79 | Ceanothine D | Peptide |
| 52 | 10.49 | (M+NH_4_)^+^ | 198.0638 | 216.0976 | C_8_H_10_N_2_O_4_ | 86.98 | 1.30 | Clavaminic acid **^d^** | Fatty acid |
| 53 | 9.56 | (M+H)^+^ | 400.1738 | 401.1812 | C_19_H_28_O_9_ | 90.98 | -1.14 | Corchoionoside B | Secondary metabolite |
| 54 | 13.17 | (M+NH_4_)^+^ | 446.1804 | 464.2145 | C_20_H_30_O_11_ | 89.90 | -3.52 | Crosatoside B | Oligosaccharide |
| 55 | 10.29 | (M+Na)^+^ | 956.5322 | 979.5213 | C_46_H_72_N_10_O_12_ | 91.54 | 1.00 | Cyanopeptolin A | Peptide |
| 56 | 11.10 | (M+H)^+^ | 204.0901 | 205.0972 | C_11_H_12_N_2_O_2_ | 96.43 | -1.09 | Ethotoin **^b^** | Secondary metabolite |
| 57 | 8.70 | (M+NH_4_)^+^ | 522.2594 | 540.2935 | C_31_H_38_O_7_ | 89.58 | 4.48 | Exiguaflavanone D | Secondary metabolite |
| 58 | 14.51 | (M+H)^+^ | 174.0643 | 175.0716 | C_6_H_10_N_2_O_4_ | 98.36 | -1.52 | Formylisoglutamine **^a^** | Peptide |
| 59 | 8.69 | (M+NH_4_)^+^ | 287.1260 | 305.1597 | C_15_H_17_N_3_O_3_ | 94.71 | 3.55 | Gabazine / SR95531 **^d^** | Secondary metabolite |
| 60 | 14.12 | (M+NH_4_)^+^ | 516.3064 | 534.3404 | C_30_H_44_O_7_ | 87.68 | 4.38 | Ganoderenic acid C **^b^** | Secondary metabolite |
| 61 | 11.24 | (M+H)^+^ | 285.1692 | 286.1766 | C_13_H_23_N_3_O_4_ | 82.67 | -1.16 | Gly Pro Leu | Peptide |
| 62 | 8.34 | (M+H)^+^ | 625.3439 | 626.3514 | C_32_H_51_NO_11_ | 90.85 | 3.66 | Glycochenodeoxycholic acid 3-glucuronide | Fatty acid |
| 63 | 8.29 | (M+H)^+^ | 417.2017 | 418.2086 | C_20_H_27_N_5_O_5_ | 96.26 | -1.17 | His Tyr Val | Peptide |
| 64 | 8.37 | (M+NH_4_)^+^ | 254.1379 | 272.1717 | C_11_H_18_N_4_O_3_ | 99.26 | 0.11 | Histidinyl-Valine **^d^** | Peptide |

**Supplementary Table 2. (continued)**

| **No.** | **RT (min)** | **Ion** | **Mass** | **m/z** | **Molecular formula** | **Score** | **DB differences (ppm)** | **Tentative identified metabolites** | **Metabolites Classification** |
| --- | --- | --- | --- | --- | --- | --- | --- | --- | --- |
| 65 | 7.73 | (M+NH_4_)^+^ | 169.1102 | 187.1440 | C_9_H_15_NO_2_ | 87.48 | 0.73 | Homoarecoline | Secondary metabolite |
| 66 | 13.56 | (M+H)^+^ | 430.2578 | 431.2657 | C_23_H_34_N_4_O_4_ | 88.00 | 0.54 | Ile Leu Trp | Peptide |
| 67 | 11.90 | (M+H)^+^ | 358.2580 | 359.2661 | C_17_H_34_N_4_O_4_ | 97.40 | -2.19 | Ile Lys Val | Peptide |
| 68 | 7.86 | (M+NH_4_)^+^ | 228.1474 | 246.1817 | C_11_H_20_N_2_O_3_ | 85.57 | -2.36 | Ile Pro | Peptide |
| 69 | 8.44 | (M+H)^+^ | 301.1426 | 302.1505 | C_16_H_19_N_3_O_3_ | 82.74 | -2.76 | Isofebrifugine | Secondary metabolite |
| 70 | 10.23 | (M+H)^+^ | 210.1368 | 211.1445 | C_11_H_18_N_2_O_2_ | 86.21 | -1.62 | L,L-Cyclo(leucylprolyl) **^a^** | Peptide |
| 71 | 8.11 | (M+H)^+^ | 196.1212 | 197.1285 | C_10_H_16_N_2_O_2_ | 93.72 | -0.02 | L-alpha-Amino-1H-pyrrole-1-hexanoic acid **^d^** | Peptide |
| 72 | 10.94 | (M+H)^+^ | 264.1844 | 265.1918 | C_15_H_24_N_2_O_2_ | 97.03 | -2.40 | Lamprolobine | Secondary metabolite |
| 73 | 11.10 | (M+Na)^+^ | 335.1848 | 358.1740 | C_17_H_25_N_3_O_4_ | 99.73 | -0.77 | Leu Gly Phe | Peptide |
| 74 | 9.89 | (M+H)^+^ | 400.2803 | 401.2876 | C_18_H_36_N_6_O_4_ | 99.19 | -1.28 | Leu Ile Arg | Peptide |
| 75 | 9.56 | (M+H)^+^ | 445.2673 | 446.2745 | C_23_H_35_N_5_O_4_ | 93.60 | 3.69 | Leu Trp Lys | Peptide |
| 76 | 10.94 | (M+H)^+^ | 327.2164 | 328.2238 | C_16_H_29_N_3_O_4_ | 96.61 | -1.84 | Leu Val Pro | Peptide |
| 77 | 10.25 | (M+H)^+^ | 259.1900 | 260.1972 | C_12_H_25_N_3_O_3_ | 86.02 | -1.55 | Leucyl-Lysine **^b^** | Peptide |
| 78 | 13.98 | (M+H)^+^ | 317.1740 | 318.1813 | C_17_H_23_N_3_O_3_ | 99.83 | -0.32 | Leucyl-Tryptophan **^a^** | Peptide |
| 79 | 7.43 | (M+H)^+^ | 262.1318 | 263.1389 | C_14_H_18_N_2_O_3_ | 94.21 | -0.14 | L-prolyl-L-phenylalanine | Peptide |
| 80 | 8.97 | (M+H)^+^ | 255.1583 | 256.1659 | C_12_H_21_N_3_O_3_ | 93.88 | 0.01 | L-Pyrrolysine | Peptide |
| 81 | 8.44 | (M+H)^+^ | 293.1744 | 294.1817 | C_15_H_23_N_3_O_3_ | 85.69 | -1.69 | Lysyl-Phenylalanine **^d^** | Peptide |
| 82 | 8.71 | (M+H)^+^ | 243.1586 | 244.1659 | C_11_H_21_N_3_O_3_ | 85.35 | -1.31 | Lysyl-Proline | Peptide |
| 83 | 3.16 | (M+Na)^+^ | 245.1739 | 268.1637 | C_11_H_23_N_3_O_3_ | 84.83 | 0.18 | Lysyl-Valine | Peptide |
| 84 | 13.56 | (M+H)^+^ | 198.1009 | 199.1084 | C_9_H_14_N_2_O_3_ | 80.20 | -2.41 | Metharbital | Secondary metabolite |
| 85 | 12.98 | (M+Na)^+^ | 800.4929 | 823.4826 | C_42_H_72_O_14_ | 97.74 | -0.91 | Momordicoside C | Secondary metabolite |

**Supplementary Table 2. (continued)**

| **No.** | **RT (min)** | **Ion** | **Mass** | **m/z** | **Molecular formula** | **Score** | **DB differences (ppm)** | **Tentative identified metabolites** | **Metabolites Classification** |
| --- | --- | --- | --- | --- | --- | --- | --- | --- | --- |
| 86 | 7.33 | (M+H)^+^ | 434.1926 | 435.2001 | C_23_ H_30_ O_8_ | 91.21 | 3.48 | Myristicanol A | Secondary metabolite |
| 87 | 8.27 | (M+H)^+^ | 203.0792 | 204.0864 | C_8_ H_13_ N O_5_ | 98.87 | 0.87 | N2-Acetyl-L-aminoadipate **^d^** | Peptide |
| 88 | 13.16 | (M+NH_4_)^+^ | 246.1015 | 264.1353 | C_13_ H_14_ N_2_ O_3_ | 82.97 | -4.11 | N-Acetyl-D-tryptophan | Peptide |
| 89 | 13.96 | (M+H)^+^ | 176.0953 | 177.1025 | C_10_ H_12_ N_2_ O | 99.16 | -1.64 | N-Hydroxyl-tryptamine **^d^** | Secondary metabolite |
| 90 | 8.99 | (M+H)^+^ | 168.0685 | 169.0758 | C_11_ H_8_ N_2_ | 85.91 | 1.35 | Norharman | Secondary metabolite |
| 91 | 14.76 | (M+Na)^+^ | 834.4990 | 857.4884 | C_42_ H_74_ O_16_ | 96.65 | -1.52 | Notoginsenoside J | Secondary metabolite |
| 92 | 14.49 | (M+NH_4_)^+^ | 157.0852 | 175.1191 | C_6_H_11_N_3_O_2_ | 85.39 | -0.31 | O(2)-vinyl-1-(pyrrolidin-1-yl)  diazen-1-ium-1,2-diolate  (V-PYRRO/NO) **^d^** | Secondary metabolite |
| 93 | 9.01 | (M+NH_4_)^+^ | 439.2225 | 457.2564 | C_22_H_33_NO_8_ | 91.78 | -4.27 | Parsonsine | Secondary metabolite |
| 94 | 9.65 | (M+H)^+^ | 234.1367 | 235.1440 | C_13_H_18_N_2_O_2_ | 84.98 | 0.49 | p-Coumaroylputrescine | Secondary metabolite |
| 95 | 7.83 | (M+H)^+^ | 434.2629 | 435.2702 | C_21_H_34_N_6_O_4_ | 94.66 | 2.85 | Phe Arg Leu | Peptide |
| 96 | 10.47 | (M+H)^+^ | 392.2067 | 393.2138 | C_19_H_28_N_4_O_5_ | 93.47 | -1.87 | Phe Asn Ile | Peptide |
| 97 | 8.11 | (M+H)^+^ | 350.1957 | 351.2027 | C_17_H_26_N_4_O_4_ | 93.83 | -0.73 | Phe Lys Gly **^b^** | Peptide |
| 98 | 10.94 | (M+H)^+^ | 406.2588 | 407.2661 | C_21_H_34_N_4_O_4_ | 98.34 | -1.86 | Phe Lys Leu **^b^** | Peptide |
| 99 | 13.56 | (M+H)^+^ | 379.1751 | 380.1824 | C_18_H_25_ N_3_ O_6_ | 98.22 | -1.91 | Phe Val Asp | Peptide |
| 100 | 10.94 | (M+H)^+^ | 361.2004 | 362.2077 | C_19_H_27_N_3_O_4_ | 99.59 | -0.75 | Phe Val Pro | Peptide |
| 101 | 13.65 | (M+H)^+^ | 220.1222 | 221.1295 | C_12_H_16_N_2_O_2_ | 94.42 | -4.60 | Phenylacetylglycine  dimethylamide | Peptide |
| 102 | 11.08 | (M+H)^+^ | 882.5215 | 883.5290 | C_47_H_79_O_13_P | 84.30 | 4.89 | PI(18:1(9Z)/20:5  (5Z,8Z,11Z,14Z,17Z)) | Fatty acid |

**Supplementary Table 2. (continued)**

| **No.** | **RT (min)** | **Ion** | | **Mass** | | **m/z** | | **Molecular formula** | | **Score** | | **DB differences (ppm)** | | **Tentative identified metabolites** | | **Metabolites Classification** | |
| --- | --- | --- | --- | --- | --- | --- | --- | --- | --- | --- | --- | --- | --- | --- | --- | --- | --- |
| 103 | 10.85 | (M+H)^+^ | | 904.5140 | | 905.5216 | | C_49_H_77_O_13_P | | 84.72 | | -4.17 | | PI(20:4(5Z,8Z,11Z,14Z)/  20:5(5Z,8Z,11Z,14Z,17Z)) | | Fatty acid | |
| 104 | 9.31 | (M+Na)^+^ | | 415.1847 | | 438.1739 | | C_20_H_25_N_5_O_5_ | | 81.98 | | 2.02 | | Pro Asn Trp | | Peptide | |
| 105 | 8.12 | (M+NH_4_)^+^ | | 371.2162 | | 389.2500 | | C_16_H_29_N_5_O_5_ | | 98.71 | | 1.80 | | Pro Gln Lys **^d^** | | Peptide | |
| 106 | 10.07 | (M+H)^+^ | | 356.2427 | | 357.2499 | | C_17_H_32_N_4_O_4_ | | 99.32 | | -0.93 | | Pro Ile Lys | | Peptide | |
| 107 | 13.32 | (M+H)^+^ | | 340.2115 | | 341.2189 | | C_16_H_28_N_4_O_4_ | | 82.77 | | -1.44 | | Pro Pro Lys **^a^** | | Peptide | |
| 108 | 12.64 | (M+H)^+^ | | 214.1315 | | 215.1389 | | C_10_H_18_N_2_O_3_ | | 98.02 | | 1.20 | | Prolyl-Valine | | Peptide | |
| 109 | 11.46 | (M+H)^+^ | | 129.0584 | | 130.0657 | | C_9_H_7_N | | 82.10 | | -4.49 | | Quinoline | | Secondary metabolite | |
| 110 | 14.24 | (M+H)^+^ | | 388.2097 | | 389.2171 | | C_19_H_32_O_8_ | | 98.24 | | 0.16 | | Rehmaionoside C | | Secondary metabolite | |
| 111 | 19.59 | (M+H)^+^ | | 122.1096 | | 123.1170 | | C_9_H_14_ | | 83.96 | | -0.56 | | Santene **^a^** | | Secondary metabolite | |
| 112 | 8.28 | (M+H)^+^ | | 291.0946 | | 292.1019 | | C_11_H_17_NO_8_ | | 96.94 | | 2.76 | | Sarmentosin epoxide | | Oligosaccharide | |
| 113 | 8.29 | (M+H)^+^ | | 494.2167 | | 495.2237 | | C_25_H_34_O_10_ | | 88.82 | | -3.08 | | Soularubinone | | Secondary metabolite | |
| 114 | 13.96 | (M+H)^+^ | | 428.2043 | | 429.2117 | | C_21_H_32_O_9_ | | 99.39 | | 0.69 | | Taraxacolide 1-O-b-D-  glucopyranoside | | Secondary metabolite | |
| 115 | 14.27 | (M+H)^+^ | | 464.2433 | | 465.2502 | | C_26_H_32_N_4_O_4_ | | 87.91 | | -2.00 | | Trp Ile Phe | | Peptide | |
| 116 | 10.07 | (M+H)^+^ | | 358.1642 | | 359.1716 | | C_18_H_22_N_4_O_4_ | | 95.09 | | -0.24 | | Trp Pro Gly | | Peptide | |
| 117 | 13.56 | (M+H)^+^ | | 416.2424 | | 417.2500 | | C_22_H_32_N_4_O_4_ | | 95.03 | | -0.08 | | Trp Val Ile | | Peptide | |
| 118 | 8.34 | (M+NH_4_)^+^ | | 351.1801 | | 369.2140 | | C_17_H_25_N_3_O_5_ | | 83.67 | | -2.07 | | Tyr Gly Leu | | Peptide | |
| 119 | 7.27 | (M+H)^+^ | | 420.2494 | | 421.2566 | | C_20_H_32_N_6_O_4_ | | 98.10 | | -2.04 | | Val Arg Phe | | Peptide | |
| 120 | 13.96 | (M+H)^+^ | | 204.0891 | | 205.0963 | | C_11_H_12_N_2_O_2_ | | 95.87 | | 3.74 | | Vasicinol | | Secondary metabolite | |
| 121 | 8.20 | (M+H)^+^ | 199.1317 | | 200.1391 | | C_9_H_17_N_3_O_2_ | | 83.43 | | 1.70 | | Vinyl-L-NIO | | Secondary metabolite | |  |

**Supplementary Table 2. (continued)**

| **No.** | | **RT (min)** | | **Ion** | **Mass** | | **m/z** | | **Molecular formula** | | **Score** | | **DB differences (ppm)** | | **Tentative identified metabolites** | | **Metabolites Classification** |
| --- | --- | --- | --- | --- | --- | --- | --- | --- | --- | --- | --- | --- | --- | --- | --- | --- | --- |
| **Cluster 3 (Perlis, Kelantan, Kedah and Penang)** | | | | | | | | | | | | | | | | | |
| 1 | | 14.51 | | (M+H)^+^ | 156.0538 | | 157.0611 | | C_6_H_8_N_2_O_3_ | | 84.94 | | -1.96 | | (S)-3-(Imidazol-5-yl)lactate **^d^** | | Oligosaccharide |
| 2 | | 3.71 | | (M+H)^+^ | 129.0902 | | 130.0974 | | C_5_H_11_N_3_O | | 87.30 | | 0.31 | | (S)-Piperazine-2-carboxamide **^d^** | | Oligosaccharide |
| 3 | | 9.45 | | (M+H)^+^ | 291.0956 | | 292.1028 | | C_11_H_17_NO_8_ | | 99.07 | | -0.71 | | 2,7-Anhydro-alpha-N-acetylneuraminic acid **^d^** | | Oligosaccharide |
| 4 | 7.24 | | (M+NH_4_)^+^ | | | 118.0423 | | 136.0760 | | C_8_H_6_O | | 97.60 | | -3.48 | | 3,5,7-Octatriyn-1-ol **^d^** | Fatty acid |
| 5 | 3.71 | | (M+H)^+^ | | | 115.0634 | | 116.0706 | | C_5_H_9_NO_2_ | | 98.39 | | -0.94 | | 3-Acetamidopropanal **^d^** | Oligosaccharide |
| 6 | 8.13 | | (M+H)^+^ | | | 139.0747 | | 140.0820 | | C_6_H_9_N_3_O | | 86.34 | | -0.92 | | 4-Amino-5-hydroxymethyl-2-methylpyrimidine **^a^** | Nucleotide |
| 7 | 7.95 | | (M+NH_4_)^+^ | | | 208.1216 | | 226.1554 | | C_11_H_16_N_2_O_2_ | | 85.73 | | -2.11 | | 4-Dimethylamino-L-phenylalanine **^c^** | Peptide |
| 8 | 7.96 | | (M+H)^+^ | | | 168.0902 | | 169.0972 | | C_8_H_12_N_2_O_2_ | | 92.47 | | -1.89 | | 5-(4-Piperidyl)isoxazol-3-ol / 4-PIOL **^c^** | Secondary metabolite |
| 9 | 9.11 | | (M+H)^+^ | | | 218.1422 | | 219.1494 | | C_13_H_18_N_2_O | | 99.01 | | -1.18 | | 5-Methoxydimethyltryptamine **^d^** | Secondary metabolite |
| 10 | 14.27 | | (M+H)^+^ | | | 248.1160 | | 249.1236 | | C_13_H_16_N_2_O_3_ | | 91.44 | | 0.51 | | 6-Hydroxymelatonin **^d^** | Secondary metabolite |
| 11 | 13.97 | | (M+H)^+^ | | | 388.2115 | | 389.2190 | | C_20_H_28_N_4_O_4_ | | 96.38 | | -1.24 | | Ala Trp Leu **^a^** | Peptide |
| 12 | 10.36 | | (M+H)^+^ | | | 226.1070 | | 227.1143 | | C_9_H_14_N_4_O_3_ | | 86.50 | | -1.96 | | Alanyl-Histidine | Peptide |
| 13 | 14.50 | | (M+H)^+^ | | | 186.1005 | | 187.1079 | | C_8_H_14_N_2_O_3_ | | 97.88 | | -0.46 | | Alanyl-Proline **^d^** | Peptide |
| 14 | 13.94 | | (M+H)^+^ | | | 232.1580 | | 233.1653 | | C_14_H_20_N_2_O | | 98.61 | | -1.86 | | Albine **^a^** | Secondary metabolite |
| 15 | 7.97 | | (M+NH_4_)^+^ | | | 141.0790 | | 159.1128 | | C_7_H_11_NO_2_ | | 87.85 | | 0.07 | | Arecaidine **^d^** | Secondary metabolite |
| 16 | 3.72 | | (M+H)^+^ | | | 287.1961 | | 288.2034 | | C_12_H_25_N_5_O_3_ | | 99.29 | | -1.34 | | Arginyl-Leucine | Peptide |
| 17 | 12.13 | | (M+H)^+^ | | | 271.1653 | | 272.1725 | | C_11_H_21_N_5_O_3_ | | 84.19 | | -3.04 | | Arginyl-Proline **^d^** | Peptide |
| 18 | 7.16 | | (M+H)^+^ | | | 111.0798 | | 112.0870 | | C_5_H_9_N_3_ | | 87.14 | | -1.03 | | Betazole **^d^** | Secondary metabolite |

**Supplementary Table 2. (continued)**

| **No.** | **RT (min)** | **Ion** | **Mass** | **m/z** | **Molecular formula** | **Score** | **DB differences (ppm)** | **Tentative identified metabolites** | **Metabolites Classification** |
| --- | --- | --- | --- | --- | --- | --- | --- | --- | --- |
| 19 | 7.05 | (M+H)^+^ | 109.0642 | 110.0714 | C_5_H_7_N_3_ | 86.96 | -1.59 | Brunfelsamidine **^c^** | Secondary metabolite |
| 20 | 10.30 | (M+NH_4_)^+^ | 198.0642 | 216.0982 | C_8_H_10_N_2_O_4_ | 95.18 | -0.81 | Clavaminic acid **^d^** | Fatty acid |
| 21 | 14.51 | (M+H)^+^ | 174.0643 | 175.0716 | C_6_H_10_N_2_O_4_ | 98.36 | -1.52 | Formylisoglutamine **^a^** | Peptide |
| 22 | 8.51 | (M+NH_4_)^+^ | 287.1275 | 305.1612 | C_15_H_17_N_3_O_3_ | 95.38 | -1.86 | Gabazine / SR95531 **^d^** | Secondary metabolite |
| 23 | 8.20 | (M+H)^+^ | 254.1386 | 255.1459 | C_11_H_18_N_4_O_3_ | 81.57 | -2.99 | Histidinyl-Valine **^d^** | Peptide |
| 24 | 10.48 | (M+H)^+^ | 210.1375 | 211.1447 | C_11_H_18_N_2_O_2_ | 83.67 | -3.21 | L,L-Cyclo(leucylprolyl) **^a^** | Peptide |
| 25 | 7.96 | (M+H)^+^ | 196.1212 | 197.1286 | C_10_H_16_N_2_O_2_ | 98.57 | -0.24 | L-alpha-Amino-1H-pyrrole-1-hexanoic acid **^d^** | Peptide |
| 26 | 13.98 | (M+H)^+^ | 317.1740 | 318.1813 | C_17_H_23_N_3_O_3_ | 99.83 | -0.32 | Leucyl-Tryptophan **^a^** | Peptide |
| 27 | 10.73 | (M+H)^+^ | 293.1745 | 294.1815 | C_15_H_23_N_3_O_3_ | 94.08 | -1.75 | Lysyl-Phenylalanine **^d^** | Peptide |
| 28 | 7.25 | (M+H)^+^ | 503.2748 | 504.2821 | C_24_H_41_NO_10_ | 93.47 | -3.46 | Mycalamide A **^c^** | Oligosaccharide |
| 29 | 8.17 | (M+H)^+^ | 203.0798 | 204.0870 | C_8_H_13_NO_5_ | 98.57 | -1.88 | N2-Acetyl-L-aminoadipate **^d^** | Peptide |
| 30 | 14.23 | (M+H)^+^ | 176.0956 | 177.1029 | C_10_H_12_N_2_O | 85.54 | -3.63 | N-Hydroxyl-tryptamine **^d^** | Secondary metabolite |
| 31 | 14.49 | (M+NH_4_)^+^ | 157.0852 | 175.1191 | C_6_H_11_N_3_O_2_ | 85.39 | -0.31 | O(2)-vinyl-1-(pyrrolidin-1-yl)diazen-1-ium-1,2-diolate / V-PYRRO/NO **^d^** | Secondary metabolite |
| 32 | 14.33 | (M+H)^+^ | 220.1216 | 221.1289 | C_12_H_16_N_2_O_2_ | 99.06 | -2.03 | Phenylacetylglycine dimethylamide **^c^** | Peptide |
| 33 | 7.97 | (M+NH_4_)^+^ | 371.2169 | 389.2508 | C_16_H_29_N_5_O_5_ | 99.68 | -0.34 | Pro Gln Lys **^d^** | Peptide |
| 34 | 10.46 | (M+H)^+^ | 340.2100 | 341.2173 | C_16_H_28_N_4_O_4_ | 96.05 | 2.97 | Pro Pro Lys **^a^** | Peptide |
| 35 | 20.42 | (M+H)^+^ | 122.1095 | 123.1169 | C_9_H_14_ | 84.06 | 0.27 | Santene **^a^** | Secondary metabolite |
| 36 | 13.96 | (M+H)^+^ | 244.1579 | 245.1652 | C_15_H_20_N_2_O | 82.07 | -1.18 | Sophoramine | Secondary metabolite |

**Supplementary Table 2. (continued)**

| **No.** | **RT (min)** | **Ion** | **Mass** | **m/z** | **Molecular formula** | **Score** | **DB differences (ppm)** | **Tentative identified metabolites** | **Metabolites Classification** |
| --- | --- | --- | --- | --- | --- | --- | --- | --- | --- |
| **Cluster 4 (Johor, Sabah, Pahang)** | | | | | | | | | |
| 1 | 7.98 | (M+H)^+^ | 494.2172 | 495.2242 | C_25_H_34_O_10_ | 86.70 | -4.03 | (8S,8'S)-Secoisolariciresinol 9-xyloside | Oligosaccharide |
| 2 | 14.51 | (M+H)^+^ | 156.0538 | 157.0611 | C_6_H_8_N_2_O_3_ | 84.94 | -1.96 | (S)-3-(Imidazol-5-yl)lactate **^d^** | Oligosaccharide |
| 3 | 3.50 | (M+H)^+^ | 129.0903 | 130.0975 | C_5_H_11_N_3_O | 87.47 | -0.40 | (S)-Piperazine-2-carboxamide **^d^** | Oligosaccharide |
| 4 | 8.02 | (M+H)^+^ | 365.1324 | 366.1397 | C_14_H_23_NO_10_ | 99.65 | -0.53 | 2-(acetylamino)-1,5-anhydro-2-deoxy-4-O-b-D-galactopyranosyl-D-arabino-Hex-1-enitol **^b^** | Oligosaccharide |
| 5 | 7.99 | (M+H)^+^ | 291.0958 | 292.1031 | C_11_H_17_NO_8_ | 99.32 | -1.39 | 2,7-Anhydro-alpha-N-acetylneuraminic acid **^d^** | Oligosaccharide |
| 6 | 8.33 | (M+H)^+^ | 190.1110 | 191.1183 | C_11_H_14_N_2_O | 98.10 | -2.19 | 2-Methyl-5-hydroxytryptamine **^b^** | Secondary metabolite |
| 7 | 7.24 | (M+NH_4_)^+^ | 118.0423 | 136.0760 | C_8_H_6_O | 97.60 | -3.48 | 3,5,7-Octatriyn-1-ol **^d^** | Fatty acid |
| 8 | 10.29 | (M+NH_4_)^+^ | 129.0790 | 147.1128 | C_6_H_11_NO_2_ | 98.00 | -0.50 | 3-acetamidobutanal | Oligosaccharide |
| 9 | 6.95 | (M+H)^+^ | 115.0632 | 116.0706 | C_5_H_9_NO_2_ | 98.03 | 0.97 | 3-Acetamidopropanal **^d^** | Oligosaccharide |
| 10 | 11.20 | (M+H)^+^ | 166.0268 | 167.0341 | C_8_H_6_O_4_ | 87.16 | -1.15 | 3-Formylsalicylic acid **^b^** | Secondary metabolite |
| 11 | 7.78 | (M+NH_4_)^+^ | 208.1216 | 226.1554 | C_11_H_16_N_2_O_2_ | 86.36 | -2.07 | 4-Dimethylamino-L-phenylalanine **^c^** | Peptide |
| 12 | 7.17 | (M+H)^+^ | 135.0688 | 136.0759 | C_8_H_9_NO | 95.00 | -2.79 | 5-(2-Furanyl)-3,4-dihydro-2H-pyrrole **^b^** | Secondary metabolite |
| 13 | 7.78 | (M+H)^+^ | 168.0899 | 169.0972 | C_8_H_12_N_2_O_2_ | 87.57 | -0.37 | 5-(4-Piperidyl)isoxazol-3-ol / 4-PIOL **^c^** | Secondary metabolite |
| 14 | 6.95 | (M+H)^+^ | 218.1423 | 219.1496 | C_13_H_18_N_2_O | 86.74 | -1.89 | 5-Methoxydimethyltryptamine **^d^** | Secondary metabolite |
| 15 | 14.09 | (M+H)^+^ | 248.1161 | 249.1238 | C_13_H_16_N_2_O_3_ | 87.95 | 0.04 | 6-Hydroxymelatonin **^d^** | Secondary metabolite |

**Supplementary Table 2. (continued)**

| **No.** | **RT (min)** | **Ion** | **Mass** | **m/z** | **Molecular formula** | **Score** | **DB differences (ppm)** | **Tentative identified metabolites** | **Metabolites Classification** |
| --- | --- | --- | --- | --- | --- | --- | --- | --- | --- |
| 16 | 6.77 | (M+H)^+^ | 392.2175 | 393.2248 | C_18_H_28_N_6_O_4_ | 98.85 | -0.64 | Ala Phe Arg **^b^** | Peptide |
| 17 | 14.11 | (M+H)^+^ | 186.1003 | 187.1080 | C_8_H_14_N_2_O_3_ | 90.06 | 0.54 | Alanyl-Proline **^d^** | Peptide |
| 18 | 14.05 | (M+H)^+^ | 174.1117 | 175.1190 | C_6_H_14_N_4_O_2_ | 82.67 | -0.13 | Amino acid (Arg-) | Peptide |
| 19 | 7.79 | (M+NH_4_)^+^ | 141.0789 | 159.1128 | C_7_H_11_NO_2_ | 87.60 | -0.10 | Arecaidine **^d^** | Secondary metabolite |
| 20 | 2.82 | (M+H)^+^ | 289.1379 | 290.1452 | C_10_H_19_N_5_O_5_ | 83.36 | 1.98 | Arginyl-Aspartate **^b^** | Peptide |
| 21 | 11.94 | (M+H)^+^ | 271.1648 | 272.1722 | C_11_H_21_N_5_O_3_ | 85.37 | -2.07 | Arginyl-Proline **^d^** | Peptide |
| 22 | 3.50 | (M+H)^+^ | 111.0797 | 112.0871 | C_5_H_9_N_3_ | 83.04 | -2.74 | Betazole **^d^** | Secondary metabolite |
| 23 | 12.30 | (M+H)^+^ | 273.0859 | 274.0930 | C_12_H_19_NO_2_S_2_ | 82.65 | -0.61 | Brugine **^b^** | Secondary metabolite |
| 24 | 7.05 | (M+H)^+^ | 109.0642 | 110.0714 | C_5_H_7_N_3_ | 86.96 | -1.59 | Brunfelsamidine **^c^** | Secondary metabolite |
| 25 | 10.15 | (M+NH_4_)^+^ | 198.0642 | 216.0982 | C_8_H_10_N_2_O_4_ | 92.92 | -0.85 | Clavaminic acid **^d^** | Fatty acid |
| 26 | 6.96 | (M+H)^+^ | 141.0902 | 142.0975 | C_6_H_11_N_3_O | 87.94 | -0.17 | DL-Histidinol | Peptide |
| 27 | 13.75 | (M+H)^+^ | 204.0899 | 205.0972 | C_11_H_12_N_2_O_2_ | 96.50 | -1.01 | Ethotoin **^b^** | Secondary metabolite |
| 28 | 8.36 | (M+NH_4_)^+^ | 287.1273 | 305.1611 | C_15_H_17_N_3_O_3_ | 96.63 | -1.23 | Gabazine / SR95531 **^d^** | Secondary metabolite |
| 29 | 13.74 | (M+NH_4_)^+^ | 516.3062 | 534.3403 | C_30_H_44_O_7_ | 84.57 | 4.85 | Ganoderenic acid C **^b^** | Secondary metabolite |
| 30 | 7.98 | (M+H)^+^ | 254.1385 | 255.1457 | C_11_H_18_N_4_O_3_ | 95.30 | -2.24 | Histidinyl-Valine **^d^** | Peptide |
| 31 | 13.73 | (M+H)^+^ | 317.1742 | 318.1815 | C_17_H_23_N_3_O_3_ | 99.64 | -0.70 | Isoleucyl-Tryptophan | Peptide |
| 32 | 7.78 | (M+H)^+^ | 196.1212 | 197.1287 | C_10_H_16_N_2_O_2_ | 85.00 | -0.80 | L-alpha-Amino-1H-pyrrole-1-hexanoic acid **^d^** | Peptide |
| 33 | 9.13 | (M+H)^+^ | 259.1899 | 260.1972 | C_12_H_25_N_3_O_3_ | 85.58 | -1.36 | Leucyl-Lysine **^b^** | Peptide |
| 34 | 10.48 | (M+H)^+^ | 293.1743 | 294.1815 | C_15_H_23_N_3_O_3_ | 98.38 | -1.28 | Lysyl-Phenylalanine **^d^** | Peptide |
| 35 | 7.08 | (M+H)^+^ | 503.2746 | 504.2820 | C_24_H_41_NO_10_ | 94.17 | -3.13 | Mycalamide A **^c^** | Oligosaccharide |
| 36 | 8.27 | (M+H)^+^ | 203.0792 | 204.0864 | C_8_H_13_NO5 | 98.87 | 0.87 | N2-Acetyl-L-aminoadipate **^d^** | Peptide |

**Supplementary Table 2. (continued)**

| **No.** | **RT (min)** | **Ion** | **Mass** | **m/z** | **Molecular formula** | **Score** | **DB differences (ppm)** | **Tentative identified metabolites** | **Metabolites Classification** |
| --- | --- | --- | --- | --- | --- | --- | --- | --- | --- |
| 37 | 13.46 | (M+H)^+^ | 176.0953 | 177.1025 | C_10_H_12_N_2_O | 86.77 | -1.78 | N-Hydroxyl-tryptamine **^d^** | Secondary metabolite |
| 38 | 3.49 | (M+NH_4_)^+^ | 157.0852 | 175.1192 | C_6_H_11_N_3_O_2_ | 87.08 | -0.28 | O(2)-vinyl-1-(pyrrolidin-1-yl)diazen-1-ium-1,2-diolate / V-PYRRO/NO **^d^** | Secondary metabolite |
| 39 | 7.79 | (M+H)^+^ | 350.1957 | 351.2028 | C_17_H_26_N_4_O_4_ | 98.86 | -0.73 | Phe Lys Gly **^b^** | Peptide |
| 40 | 10.50 | (M+H)^+^ | 406.2582 | 407.2654 | C_21_H_34_N_4_O_4_ | 99.33 | -0.41 | Phe Lys Leu **^b^** | Peptide |
| 41 | 14.09 | (M+H)^+^ | 220.1216 | 221.1288 | C_12_H_16_N_2_O_2_ | 98.91 | -1.81 | Phenylacetylglycine dimethylamide **^c^** | Peptide |
| 42 | 7.78 | (M+NH_4_)^+^ | 371.2171 | 389.2508 | C_16_H_29_N_5_O_5_ | 99.84 | -0.39 | Pro Gln Lys **^d^** | Peptide |

**^a^** The metabolites found to be similar between Cluster 2 and 3.

**^b^** The metabolites found to be similar between Cluster 2 and 4.

**^c^** The metabolites found to be similar between Cluster 3 and 4.

**^d^** The metabolites found to be similar among Cluster 2, 3 and 4.

RT = retention time; DB = database.
